# Hidden in plain sight: challenges in proteomics detection of small ORF-encoded polypeptides

**DOI:** 10.1093/femsml/uqac005

**Published:** 2022-05-14

**Authors:** Igor Fijalkowski, Patrick Willems, Veronique Jonckheere, Laure Simoens, Petra Van Damme

**Affiliations:** iRIP Unit, Laboratory of Microbiology, Department of Biochemistry and Microbiology, Ghent University, 9000 Ghent, Belgium; iRIP Unit, Laboratory of Microbiology, Department of Biochemistry and Microbiology, Ghent University, 9000 Ghent, Belgium; iRIP Unit, Laboratory of Microbiology, Department of Biochemistry and Microbiology, Ghent University, 9000 Ghent, Belgium; iRIP Unit, Laboratory of Microbiology, Department of Biochemistry and Microbiology, Ghent University, 9000 Ghent, Belgium; iRIP Unit, Laboratory of Microbiology, Department of Biochemistry and Microbiology, Ghent University, 9000 Ghent, Belgium

**Keywords:** *Salmonella* Typhimurium, sORF-encoded polypeptides (SEPs), sORF, riboproteogenomics, proteomics, *in silico* proteomics

## Abstract

Genomic studies of bacteria have long pointed toward widespread prevalence of small open reading frames (sORFs) encoding for short proteins, <100 amino acids in length. Despite the mounting genomic evidence of their robust expression, relatively little progress has been made in their mass spectrometry-based detection and various blanket statements have been used to explain this observed discrepancy. In this study, we provide a large-scale riboproteogenomics investigation of the challenging nature of proteomic detection of such small proteins as informed by conditional translation data. A panel of physiochemical properties alongside recently developed mass spectrometry detectability metrics was interrogated to provide a comprehensive evidence-based assessment of sORF-encoded polypeptide (SEP) detectability. Moreover, a large-scale proteomics and translatomics compendium of proteins produced by *Salmonella* Typhimurium (*S*. Typhimurium), a model human pathogen, across a panel of growth conditions is presented and used in support of our *in silico* SEP detectability analysis. This integrative approach is used to provide a data-driven census of small proteins expressed by *S*. Typhimurium across growth phases and infection-relevant conditions. Taken together, our study pinpoints current limitations in proteomics-based detection of novel small proteins currently missing from bacterial genome annotations.

## Teaser

Integrative in silico proteome analysis and experimental riboproteogenomic data uncovers biases in mass spectrometric based detection of sORF-encoded small proteins.

## Introduction

With rapidly growing number of sequenced genomes, manual gene annotation had to give way to automated gene prediction algorithms. Despite their utility, a growing body of evidence suggests that these methods pose a danger of propagating biases present in current annotations and may vastly underestimate the complexity of bacterial genomic architecture (Crappé *et al*. 2015, Giess *et al*. 2017, Ndah *et al*. 2017). One particularly understudied class of genomic elements is represented by small open reading frames (sORFs), generally considered as ORFs encoding proteins (equal or) smaller than 100 amino acids in length and herein referred to as sORF-encoded polypeptides or SEPs (Miravet‐Verde *et al*. 2019). Thus far, existence of actively translated sORFs has been detected in a wide variety of organisms. Besides regulatory sORFs, many sORFs have been shown to give rise to functionally relevant protein products (Park *et al*. 2010, Adams *et al*. 2021). In bacteria, SEPs have been associated with a plethora of cellular functions, including glucose uptake, antibiotic resistance and peptidoglycan synthesis to name a few (Storz *et al*. 2014, Duval and Cossart 2017, Fijalkowska *et al*. 2020). A pivotal role of SEPs in bacterial biology is also supported by the fact that sORFs have been found to be the most frequently essential genomic elements of the *Mycoplasma pneumoniae* genome (Lluch‐Senar *et al*. 2015). These properties situate complete SEP identification as an important milestone on the way to better our understanding of bacterial systems biology.

In the past, predicted ORFs were frequently penalized based on length using arbitrary cutoffs (like 100 codons) and sequence overlap with other ORFs (Dinger *et al*. 2008, Richardson and Watson 2013, Miravet‐Verde *et al*. 2019, Fijalkowska *et al*. 2020). While current annotation pipelines largely rely on sequence composition features instead of length thresholds to distinguish coding from noncoding sequences, short proteins still remain underrepresented (Warren *et al*. 2010, Samayoa *et al*. 2011). Yet, numerous novel bacterial sORFs and SEPs have recently been discovered in bacteria using phylogenetic analyses (Sberro *et al*. 2019), transcriptomics, translatomics (Venter *et al*. 2011, Baek *et al*. 2017, Hücker *et al*. 2017, Omasits *et al*. 2017, Weaver *et al*. 2019, Fuchs *et al*. 2021, Stringer *et al*. 2021) and proteomics efforts (Impens *et al*. 2017, Yuan *et al*. 2018, Miravet‐Verde *et al*. 2019), the latter omics approaches jointly referred to as riboproteogenomics (Ndah *et al*. 2017, Willems *et al*. 2020). Algorithms used for prokaryotic sORF delineation without *a priori* knowledge (Hemm *et al*. 2008, Miravet‐Verde *et al*. 2019, Venturini *et al*. 2020) suggest that sORFs may even be counted in hundreds but experimental confirmation of these data however is only limited with few high-throughput methods reported (Miravet‐Verde *et al*. 2019), and often tedious (e.g. detection via genomic tagging and immunodetection of candidate sORFs; Vanorsdel *et al*. 2018) (Weaver *et al*. 2019, Stringer *et al*. 2021). Unsurprisingly, only a handful of bacterial SEPs were functionally characterized (Impens *et al*. 2017, Yuan *et al*. 2018). Notwithstanding the considerable efforts aiming at improving bacterial genome annotations, identification of sORFs remains a considerable challenge. Recent major advances in the field can be attributed to the development of ribosome profiling (Ribo-seq), a genomic method providing a snapshot of *in vivo* translation through deep sequencing of mRNA fragments covered by the actively translated ribosome (Ingolia *et al*. 2009). Evidence of pervasive translation in previously unannotated genomic regions sparked the development of novel Ribo-seq data-dependent ORF prediction algorithms for both eukaryotic and prokaryotic genomes (Ndah *et al*. 2017, Verbruggen *et al*. 2019). Moreover, recent introduction of retapamulin-assisted ribosome profiling (Ribo-RET) allows for more precise delineation of bacterial translation start sites aiding ORF delineation (Meydan *et al*. 2019, Weaver *et al*. 2019). Continuous developments in the field of bacterial translation initiation as well as termination profiling hold great promise in facilitating further gene discovery through employment of novel antibiotics, like lefamulin or apidaecin (Ribo-Api and Ribo-Api/Pmn (puromycin); Mangano *et al*. 2020), enabling trapping of ribosomes at start and stop codons, respectively (Vazquez-Laslop *et al*. 2022). Besides, the predicted (s)ORF repertoire can be used to inform the proteomic searches of putative sORF products and facilitate their identification. However, despite notable advances in the proteogenomic identification of SEPs owed to custom peptidomics approaches (Petruschke *et al*. 2020) and the use of matching (differential) RNA-seq or ribosome profiling-inferred databases for proteomics database searching, the repertoire of mass spectrometry (MS)-confirmed SEPs is scarce (Venturini *et al*. 2020). Besides their intrinsic small size, also their possible low abundance or low stability has often been raised as potential factors obstructing MS detection (Peeters and Menschaert 2020). Moreover, more than half of predicted SEPs in *Escherichia coli* are predicted to be single-transmembrane (TM) proteins, suggesting that their hydrophobic nature and low solubility might contribute to the poor detectability of this specific class of SEPs when using proteomics as a readout (Fontaine *et al*. 2011). Existence of such hydrophobic SEPs has also been suggested in *Streptococcus* and *Enterococcus* (Ibrahim *et al*. 2007). Considering all that, it is thus surprising that no systemic analysis investigating the challenging nature of MS-based identifiability of bacterial SEPs has thus far been conducted using the wealth of available complementary omics datasets at both proteomic and genomic levels (Gray *et al*. 2022).

Here, computational analysis aided by state-of-the-art experimental riboproteogenomics data sheds light on the challenges faced by MS-based SEP discovery. For the comprehensive detection of translated (novel) (s)ORFs, complementary Ribo-seq and Ribo-RET screening was performed in the model bacterial pathogen *Salmonella enterica* subsp. *enterica* serovar Typhimurium (further referred to as *S*. Typhimurium) sampled at various growth conditions—including infection relevant conditions—selected based on complementarity expression patterns observed in published transcriptomics efforts of the Hinton lab (Kröger *et al*. 2013, Srikumar *et al*. 2015). Following the recently reported landmark census of *S*. Typhimurium small proteins identifying 139 novel high-confidence sORFs (Venturini *et al*. 2020) of which SEP expression was validated for 15 out of 16 tested candidates, we provide additional rich datasets and evaluate the utility of translation initiation profiling in refining the compendium of putative novel small proteins. Using initiating and elongating Ribo-seq signals, a new gene discovery pipeline was applied to detect and delineate translated ORFs. Moreover, matching proteomics datasets generated and analyzed using our recently published proteogenomic pipeline (Willems *et al*. 2020) further strengthened the confidence of novel proteogenomic identifications, with a specific focus on SEPs. The findings highlight the importance of proteogenomic efforts for the continuous enrichment of bacterial genome annotations (Venter *et al*. 2011, Giess *et al*. 2017, Ndah *et al*. 2017, Omasits *et al*. 2017, Fijalkowska *et al*. 2020, Willems *et al*. 2020, Fuchs *et al*. 2021).

## Materials and methods

### Bacterial culture

The *S*. Typhimurium wild-type strain SL1344 (Hoiseth and Stocker, 1981) (Genotype: hisG46, Phenotype: His(-); biotype 26i) was obtained from the *Salmonella* Genetic Stock Centre (SGSC, Calgary, Canada; cat no. 438; Hoiseth and Stocker 1981). The SL1344 has been further modified by deleting *tolC* (Δ*tolC*) using λ red-mediated recombineering as described in (Datsenko and Wanner 2000), with the following homology primer pair: CAACAAGGAATGCAAATGAAGAAATTGCTCCCCATCCTTATCGGCtgtgtaggctggagctgcttc; CCAGCGAATAACTTATCAATGCCGGAATGGATTGCCGTTATTGCTtatgaatatcctccttagttc. Bacterial growth was performed using liquid Lennox broth (LB) growth medium (10 g/L Bacto tryptone, 5 g/L Bacto yeast extract, 5 g/L NaCl), on LB agar plates (10 g/L Bacto tryptone, 5 g/L Bacto yeast extract, 5 g/L NaCl, 12 g/L agar) or variants of phosphate carbon nitrogen (PCN) medium (Löber *et al*. 2006) (lnSPI2; *Salmonella* pathogenicity island 2-inducing condition; pH 5.8, 0.4 mM Pi), low Mg^2+^ SPI2-inducing PCN (low Mg^2+^; pH 5.8, 0.4 mM Pi) containing low levels (10 μM) of magnesium sulfate (PCN medium was stored at 4°C and brought at room temperature for cultivation). Viewing the His-auxotrophic nature of the SL1344 strain used, all PCN media were supplemented with histidine to a final concentration of 5 mM.

### Shotgun proteomics sample collection and preparation

For shotgun total proteomics analysis, WT SL1344 cells were grown in various conditions: early exponential growth phase (EEP; OD600 0.1), mid-exponential growth phase (MEP; OD600 0.3), late exponential growth phase (LEP; OD600 1.0), early stationary phase (ESP, OD600 2.0) and late stationary phase (LSP, OD600 2.0 + 6 h of extra growth). Environmental shocks in LB were performed on MEP-grown bacteria as follows: osmotic shock (NaCl shock), by addition of NaCl to a final concentration of 0.3 M and continued growth for 10 min; anaerobic shock, a 50 mL culture was transferred into a prewarmed (37°C) 50-mL centrifuge tube, tightly closed and incubated for an additional 30 min at 37°C without agitation. For growth in variants of PCN minimal medium (Löber *et al*. 2006), overnight grown LB cultures (8 mL) were washed twice in PCN medium before resuspension at OD 0.02. Cells were grown in T175 mL flasks in 50-mL SPI2-inducing PCN (InSPI2; pH 5.8, 0.4 mM Pi), or low magnesium SPI2-inducing PCN (Low Mg^2+^; pH 5.8, 0.4 mM Pi) containing low levels (10 μM) of magnesium sulfate. All PCN media were supplemented with 5 mM (f.c.) histidine. The nitric oxide shock conducted in PCN (InSPI2) was performed at OD600 0.3 by addition of the nitric oxide donor-2-Spermine NONOate to a final concentration of 250 μM for 20 min (nitric oxide shock (InSPI2)). Per biological replicate sample, the equivalent of 4 × 50, 50, 10 or 5 mL of culture were sampled in case of the OD600 0.1, OD600 0.3, OD600 1.0 and OD600 2.0 growth conditions, respectively. Cell pellets were resuspended in Gu.HCl lysis buffer (4 M Gu.HCl, 50 mM NH_4_HCO_3_; pH 7.9) and subjected to three rounds of mechanical freeze–thaw lysis in liquid nitrogen followed by sonication (Branson probe sonifier output 4, 50% duty cycle, 2 × 30 s, 1 s pulses). Lysates were clarified by centrifugation for 10 min at 16 100 × *g* (4°C). Protein concentration was determined by Bradford measurement according to the manufacturer's instructions. An aliquot equivalent of 400 µg (∼2 × 10^−9^ bacteria) protein lysate was transferred to a clean tube, diluted to 2.7 mg/mL with lysis buffer, 2× diluted with HPLC grade water and precipitated with 4× volumes of −20°C acetone overnight (−20°C). The precipitated protein material was recovered by centrifugation for 15 min at 3500 × *g* (4°C), pellets washed twice with −20°C 80% acetone and air dried upside down for 10 min at RT or until no residual acetone odor remained. Pellets were resuspended in 200 µL TFE (2,2,2-trifluoroethanol) digestion buffer (10% TFE, 100 mM ammonium bicarbonate) with sonication until a homogenous suspension was reached. All samples were digested overnight at 37°C using mass spec grade Trypsin/Lys-C Mix (Promega, Madison, WI) (enzyme/substrate of 1:100 w/w) while mixing (550 rpm). Samples were acidified with TFA to a final concentration of 0.5%. Samples were cleared from insoluble particulates by centrifugation for 10 min at 16 100 × *g* (4°C) and the supernatant was transferred to clean tubes. Methionine oxidation was performed by the addition of H_2_O_2_ to reach an f.c. of 0.5% for 30 min at 30°C. Solid phase extraction of peptides was performed using C18 reversed phase sorbent containing 100 µL pipette tips (Bond Elut OMIX 100 µL C18 tips; Agilent, Santa Clara, CA) according to the manufacturer's instructions. Eluted samples were vacuum-dried in a SpeedVac concentrator (Thermo Fisher Scientific, Waltham, USA), redissolved in 2 mM tris(2-carboxyethyl)phosphine in 2% acetonitrile and injected onto the Q Exactive HF instrument (Thermo Fisher Scientific, Waltham, USA).

### Ribo-seq and Ribo-RET sample collection

Biological replicate cultures were used to obtain two total translatomes and two retapamulin-treated translatomes per condition (i.e. total of four cultures per condition). Wild-type bacteria were used for total translatome samples, while the isogenic Δ*tolC* strain was used for retapamulin-treated translatome samples. For each replicate, a single colony was picked from an LB plate, incubated in 5 mL liquid LB and grown overnight at 37°C with agitation (180 rpm). Overnight cultures were diluted 1:200 (OD600 0.02) and used to inoculate 50 mL cultures of *S*. Typhimurium grown under different growth and infection-relevant conditions. More specifically, bacteria were grown in a limited panel of conditions described earlier: MEP, LEP, NaCl shock, anaerobic shock, nitric oxide shock, InSPI2 and InSPI2 Low Mg^2+^ (Srikumar *et al*. 2015).

Following the growth conditions described earlier, 50 mL cultures (or 5 mL in case of LEP samples) were treated with either chloramphenicol (100 µg/mL) or retapamulin (10 µg/mL; 100 × MIC Δ*tolC* of 0.1 µg/mL) for 5 min at 37°C, collected using centrifugation (2500 × *g*, 3 min, 4°C) and the bacterial pellets flash frozen in liquid nitrogen and stored at −80°C until further processing.

### Total translatome and translation initiation profiling

All cell pellets were resuspended in 1 mL of lysis buffer (10 mM MgCl_2_, 100 mM NH_4_Cl, 20 mM Tris pH 8.0 20 U/mL of RNase-free DNase I (Thermo Fisher Scientific, Waltham, USA) 1 mM chloramphenicol and 20 µL/mL lysozyme) thawed and flash frozen. Subsequently, 5 mM CaCl_2_, 30 µL 10% DOC and protease inhibitor cocktail (Roche, cat# P8465) were added. The lysates were incubated on ice for 5 min and cellular debris was removed by centrifuging (16 000 × *g*, 10 min, 4°C). Total RNA content was measured using NanoDrop (Thermo Fisher Scientific, Waltham, USA; 1:100 dilutions, blank 1:100 dilution of the lysis buffer because chloramphenicol absorbs at 260 nm) and MNase (Roche, cat# 10107921001, Basel, Switzerland) digestion was performed by the addition of the enzyme (15 U/1.0 OD_260_ of RNA) and 1 h incubation at 25°C with mixing (650 RPM). Subsequently, 10 mM EGTA was added to stop the reaction. Four hundred fifty microliters of each sample was ran through a 540 µL sucrose cushion (1 M sucrose in 10 mM MgCl_2_, 100 mM NH4Cl, 20 mM Tris, pH 8.0, 1 mM chloramphenicol, 100 U/mL superasin, 2 mM DTT) by ultracentrifugation (4°C, 200 000 × *g*, 4 h, TLA-120.2 rotor). Resulting pellets were resuspended in 300 µL of lysis buffer and RNA extracted using the warm acid phenol chloroform method as previously described (Ingolia *et al*. 2009). Ribosome protected footprints were subsequently purified by excision from a 15% TBE-Urea polyacrylamide gel (Invitrogen, Waltham, USA) ran in TBE (Ambion, Waltham, USA). The gel was pre-run for 30 min at 200V. 2× sample loading buffer (TBE-Urea, Novex, Invitrogen, Waltham, USA) was added to the samples. Alongside the samples, 1 µL of 10 bp ladder (Invitrogen, Waltham, USA) and RNA oligos of 26 and 32 nt length (10 pmol) were ran for size control. All samples were denatured at 70°C for 2 min, chilled and loaded on the gel. The gel was ran for 65 min at 200 V. Subsequently, the gel was stained for 20 min (RT) with SYBR gold according to manufacturer's instructions (Invitrogen, Waltham, USA) and desired bands demarked by the control oligos were excised. RNA was recovered through isopropanol precipitation as previously described (Ingolia *et al*. 2009). All samples were resuspended in 15 µL of 10 mM Tris pH 7.0 and dephosphorylated using T4 polynucleotide kinase (NEB, Ipswitch, USA) (2 µL of enzyme per sample corresponding to 200 ng of purified footprints). The samples were incubated for 1 h at 37°C without shaking. The volume of samples was adjusted to 100 µL with nuclease free water and the samples were purified using RNA Clean & Concentrator-5 kit (Zymo Research, Irvine, USA) according to the manufacturer's instructions with the following modification; 200 µL of RNA binding buffer was used and 450 µL of 100% EtOH. Samples were eluted with 11 µL of nuclease free water and used as input for library preparation. The libraries were generated with NEXTFLEX Small RNA-seq kit v3 for Illumina platforms (PerkinElmer, Waltham, USA) according to manufacturer's instructions from 200 ng of purified, dephosphorylated footprints. Final library PCR product (∼160 nt) was assessed using Bioanalyzer 2100 High Sensitivity DNA Assay (Agilent, Santa Clara, USA) and purified from 8% TB polyacrylamide gel (Invitrogen, Waltham, USA; 45 min, 180 V) by isopropanol precipitation. Concentration of purified libraries were measured using the High Sensitivity Qubit assay (Invitrogen, Waltham, USA) and 1.8 pmol of libraries was sequenced on NextSeq 550 using the NextSeq High Output kit (v2.5, 75 cycles; Illumina, San Diego, USA).

### MS/MS analysis and data analysis

The LC-MS/MS system was composed of the Ultimate 3000 RSLC nano system (Thermo Fisher Scientific, Waltham, USA) connected to a Q Exactive HF mass spectrometer (Thermo Fisher Scientific, Waltham, USA) equipped with a Nanospray Flex Ion source (Thermo Fisher Scientific, Waltham, USA). Trapping was performed at 10 μL/min for 4 min in solvent A (0.1% formic acid in water/ACN; 2:8, v/v) on a 20 mm trapping column (made in-house, 100 μm internal diameter, 5 μm beads, C18 Reprosil-HD, Dr. Maisch, Germany) and the sample was loaded on a 200 cm long micro pillar array column (PharmaFluidics, Ghent, Belgium) with C18-endcapped functionality mounted in the UltiMate 3000’s column oven at 50°C. Fused silica PicoTip emitter (10 µm inner diameter) (New Objective, Littleton, USA) was connected to the µPAC outlet union and a grounded connection was provided to this union. Peptides were eluted by a nonlinear increase from 1% to 55% MS solvent B (0.1% formic acid in water/ACN; 2:8, v/v) over 145 min, first at a flow rate of 750 nL/min, then at 300 nL/min, followed by a 15 min wash reaching 99% MS solvent B and reequilibration with MS solvent A. The mass spectrometer was operated in data-dependent, positive ionization mode, automatically switching between MS and MS^2^ acquisition for the 16 most abundant ion peaks per MS spectrum. Full-scan MS spectra (375–1500 m/z) were acquired at a resolution of 60 000 in the Orbitrap analyzer after accumulation to a target value (AGC target) of 3 000 000. The 16 most intense ions above a threshold value of 13 000 were isolated (window of 1.5 Th) for fragmentation at a normalized collision energy of 28% after filling the trap at a target value of 100 000 for maximum 80 ms. MS^2^ spectra, with a first fixed mass of 145 m/z, were acquired at a resolution of 15 000 in the Orbitrap analyzer. The S-lens RF level was set at 50 and precursor ions with single, unassigned and >7 charge states were excluded from fragmentation selection.

Raw data files were searched using MaxQuant version 1.6.3.4 (Tyanova *et al*. 2016) and spectra searched against the ENSEMBL database for *S*. Typhimurium strain SL1344. For both proteomics and genomics datasets the *Salmonella enterica* subsp. *enterica* serovar Typhimurium str. SL1344 (enterobacteria) genome assembly ASM21085v2 from The Wellcome Trust Sanger Institute (GCA_000210855.2) containing 4672 protein entries was used. Multiplicity was set to 1, indicating that no labels were used. Furthermore, label-free quantitation (LFQ) with MaxQuant's standard settings was performed with matching between runs enabled (match time window of 1.2 min and an alignment time window of 20 min). As a fixed modification, methionine oxidation (to methionine sulfoxide) was selected. We used the enzymatic rule of Trypsin/P with a maximum of 2 missed cleavages. The main search peptide tolerance was set to 4.5 ppm and the ion trap MS/MS match tolerance was set to 0.5 Da. Peptide-to-spectrum match confidence level was set at 1% FDR with an additional minimal Andromeda score of 40 for modified peptides as these settings are most commonly used by researchers. The maximal number of modifications per peptide was set to 5. Resulting protein identifications were reported in Table S1 (Supporting Information).

Additionally, proteogenomic searches were performed on the proteomics datasets using our recently published pipeline as previously described (Willems *et al*. 2020) and new proteogenomic findings (at protein and peptide level) summarized in Table S2 (Supporting Information). In short, the pipeline relies on an iterative search strategy of cofragmenting peptides with robust confidence estimating postprocessing, incorporating advanced peptide-to-spectrum match quality scoring. For this purpose, comparison to predicted fragmentation spectra (Degroeve and Martens 2013), advanced retention time prediction (Moruz *et al*. 2010) and cross-validation with Ribo-seq data are utilized. As presented in the original work, these features assure not only deeper proteome coverage but also increased confidence in identified novel genomic elements.

### Ribosome profiling data analysis

Sequencing files were demultiplexed using bcl2fastq software (Illumina, San Diego, USA). Sample specific fastq.gz files originating from individual lanes were concatenated (cat, Unix). Unique molecule identifiers (UMI) introduced in NextFlex library were extracted from individual reads using a custom Python script. PCR bias normalization was performed using a previously published procedure (Mcglincy and Ingolia 2017). Normalized data was trimmed in two steps using cutadapt. In the first step standard Illumina adapters are removed (cutadapt -q 20 -m 25 -e 0.2) followed by secondary removal of UMIs (cutadapt -u 4 -u -4). The data are subsequently mapped to indexed rRNA sequences using Bowtie (bowtie -t -n 2 -p 6 –best). rRNA sequences were retrieved from Ensembl and GenBank. Additional sequences of tRNAs, RNA subunits of nucleoproteins and ncRNAs were also added to the rRNA index and all aligned reads were removed from further analysis. The remaining reads were mapped to the *S*. Typhimurium SL1344 genome using bowtie (-t -n 2 -p 6 -m 1 –best –strata –sam). Resulting SAM files were converted to BAM format and sorted using Samtools (Danecek *et al*. 2021). Ribosomal P-site assignment for all reads was performed using RiboWalz (Lauria *et al*. 2018) outperforming plastid (Dunn and Weissman 2016) utilized in previous studies (Fig. S1, Supporting Information) (Ndah *et al*. 2017). Positional data was counted using a custom Python script and normalized to RPM values. Positional read occupancy tables were created using Python and R tidyverse environment and exported for further analysis. Subsequently, RPKM values were calculated for all Ensembl annotated genes and separate BedGraph files for data visualization were parsed using a custom Python script and provided as supplementary material via OSF.

### Ribosome profiling data quality control

For Ribo-seq and Ribo-RET, two biological replicate samples obtained from all growth conditions (i.e. two total translatomes and two retapamulin-treated translatomes per condition) were sequenced. The ribosomal RNA (rRNA) contamination typical for Ribo-seq libraries ranged between 61% and 74% for total translatome samples. The samples were sequenced to the depth resulting in between 18 and 28 million of genome-aligned reads (after removing rRNA-aligned reads). In case of Ribo-RET, considerable rRNA contamination was detected ranging from 72% to 80% of obtained reads. The libraries were sequenced to the depth ranging from 8 to 12 million of genome aligned reads after removing rRNA-aligned reads. Sequencing statistics and metagene analysis has been summarized in Fig. S2 (Supporting Information).

### Ribosome profiling-dependent gene detection

Open reading frame (ORF) detection from Ribo-RET was performed using four distinct methods. First, a sliding window signal detection tool was devised using a custom Python script. For this tool, a 30 nt window is run through the genome aligned sample and searching for signal accumulation peaks that exceed the median RPM values of surrounding genomic positions by 3-fold. To prefilter the data, a denoising step was introduced guided by a 30 nt sliding window and removing areas of continuous low coverage (<4 reads per strand at continuous stretch of four nucleotides). Of note, the signal accumulation method detects all potential signal peaks but is severely affected by background signal producing a large number of potentially false positive hits. Despite this drawback, the sliding window method allows the annotation of start and end positions of each individual retapamulin data peak resulting from imperfect P-site assignment and thus the peak start, max, end and width data is used in subsequent ORF detection tools as an important feature.

Second, a criterium classifier was adapted from Meydan *et al*. (2019). In short, the tool assigns ribosomal density to defined initiation codons in a stepwise manner based on the probability of initiation at a given position (with annotated start sites taking priority). The tool was modified to use the SL1344 genome and Ribo-waltz corrected P-site assigned reads as inputs.

Third, a random forest classifier based on Ndah *et al*. (2017) was trained to detect initiation events based on a positive set of highly expressed (top 20%) annotated genes. A negative set of random ORFs was used to correct for false positive detection as described in the original work. The machine learning algorithm based on a modified Perl script was subsequently ran on all retapamulin samples. Resulting short sequence list reflects the positions of retapamulin initiation peaks in the input data based on signal distribution features devised from the positive training set.

Finally, a similar approach has been devised by modifying the pipeline described in Clauwaert *et al*. (2019). A neural network (NN) classifier was trained using positive datasets including the 20% highest expressed annotated genes in retapamulin samples. The NN classifier was subsequently applied to all samples providing a list of initiation events detected based on a discrete set of features.

Results from each individual ORF detection tool were parsed together into a data matrix using a custom Python script and a high confidence dataset of initiation events detected by three out of four methods cross-referenced. Subsequently, the list of initiation events was matched with a six-frame STOP to STOP database assigning most likely ORF bodies to in-frame initiation signals and selecting RET peaks as a new 5′ CDS delineator, and concomitantly as new N-terminal delineator. The resulting list of confident translated ORFs predicted based on Ribo-RET data are listed in Table S3 (Supporting Information).

### Novel ORF data analysis

The high confidence dataset (Table S3, Supporting Information) obtained was further processed using custom R and Python scripts. A differential expression analysis was performed for ORFs detected in all investigated Ribo-seq conditions using a custom R script. First, a maximal expression matrix was built to provide the oversight on all ORFs detected in at least two conditions. Such ORFs were selected for further analysis. In parallel a similar analysis was performed using corrected retapamulin peak intensity as input value in place of RPKM. Corrected retapamulin intensity values were obtained as the integral of the retapamulin peak detected normalized against detected expression within the body of the gene (background). Differential expression analysis was performed using DESeq2 package with stringent Bonferroni correction for multiple testing (Love *et al*. 2014).

## Results

### A conditional atlas of *S*.Typhimurium total protein expression

For the in-depth mapping of translated ORFs—including translated sORFs—and thus the *S*. Typhimurium translation landscape, and to understand the biases of proteomics toward SEP detection, complementary proteomics and translatomics data were generated. More specifically, translation (initiation) data originating from (retapamulin-assisted) ribosome profiling (Ribo-RET and Ribo-seq) and matching total lysate shotgun proteomics datasets were obtained from a series of representative growth conditions corresponding to various growth phases (e.g. MEP, LEP) and environmental stresses (NaCl shock, anaerobic shock, low pH, low Mg^2+^ at low pH and nitric oxide shock at low pH; see the 'Materials and Methods' section).

Conditional shotgun proteomics investigation provided a comprehensive snapshot of the annotated *S*. Typhimurium proteome, identifying 3053 out of 4672 annotated proteins (65% of the annotated proteome) with at least one unique tryptic peptide (UTP) and minimally two peptide-to-spectrum matches (PSMs) using MaxQuant (Tyanova *et al*. 2016) when searching against the Ensembl protein database (ASM21085v2; Ensembl release 51; Table S1, Supporting Information). Each growth condition was interrogated by shotgun proteomics in quadruplicates. When filtering for proteins identified in at least three replicates of at least one growth condition, 2813 protein groups remain confidently detected (92%) and quantified with an average protein peptide coverage of 43%. Owed to the number of conditions investigated and the wide dynamic range of protein expression captured (maximum intensity-based absolute quantification: iBAQ fold change of 8.9 × 10^5^), a relatively small number of proteins was uniquely identified only in one growth condition with a maximum of 14 proteins being uniquely detected in stationary phase grown cells (Table S1, Supporting Information). Proteomics evidence for the expression of 190 annotated SEPs was discovered with 170 SEPs confidently quantified (Table S1, Supporting Information). While no SEPs were exclusively identified in only one growth condition, and an average SEP peptide coverage of 44% was observed, it is noteworthy that SEPs were identified with an average of 291 PSMs compared with the average of 484 PSMs per protein identification for the entire protein dataset.

Complementary to shotgun proteomics analyses, total cellular translatomes were investigated by means of Ribo-seq. This way, 4135 genes were found to be robustly expressed (here arbitrarily defined as RPKM (reads per kilobase per million sequenced reads) value higher than 5) in at least one growth condition. The translation data positively correlates with the proteomics data (average Pearson coefficient of 0.57). When investigating general protein translation levels, a core set of 2529 robustly translated proteins (RPKM > 5) in all investigated conditions can be distinguished. Considering the RPKM cutoff of 5, a dynamic translation range of 9.3 × 10^4 ^was interrogated. The translation data was further supported by translation initiation profiling using Ribo-RET. Similar to total translatome datasets, each growth condition was sequenced in duplicates. Using an arbitrary cutoff of 32 reads at the annotated start codon we confirmed initiation events at 2799 database-annotated translation initiation sites (dbTIS). The vast majority of confirmed initiation events occur at canonical ATG codons (89%—2491 start sites), 9% (252) at GTG codons and 2% (56) at TTG codons, proportions largely in line with previously published data (Ndah *et al*. 2017, Meydan *et al*. 2019, Verbruggen *et al*. 2019) and current *S*. Typhimurium gene annotation (88% ATG, 9.2% GTG and 2.7% TTG). The summary of most commonly assigned start codons of Ribo-RET data is presented in Fig. S3 (Supporting Information).

### Integration of *in silico* proteome analysis and experimental riboproteogenomic data to uncover biases in MS-based SEP detection

The conditional *S*. Typhimurium annotated protein expression maps obtained using translatomics and proteomics were used in conjunction with *in silico* analysis to illustrate the potential biases present in proteomics detection. For this, a wide panel of chemical and biophysical property descriptors of all theoretical tryptic peptides (mass range of 600–4600 Da, length of ≥ 7 aa) resulting from *in silico* digestion of the annotated *S*. Typhimurium proteome were calculated using the propy python package (https://pypi.org/project/propy3/). Using these descriptors, we compared theoretical properties of peptides originating from database annotated SEPs with those originating from the rest of the proteome.

### SEPs detection is challenged by low numbers of MS-detectable tryptic peptides

**Figure 1.**
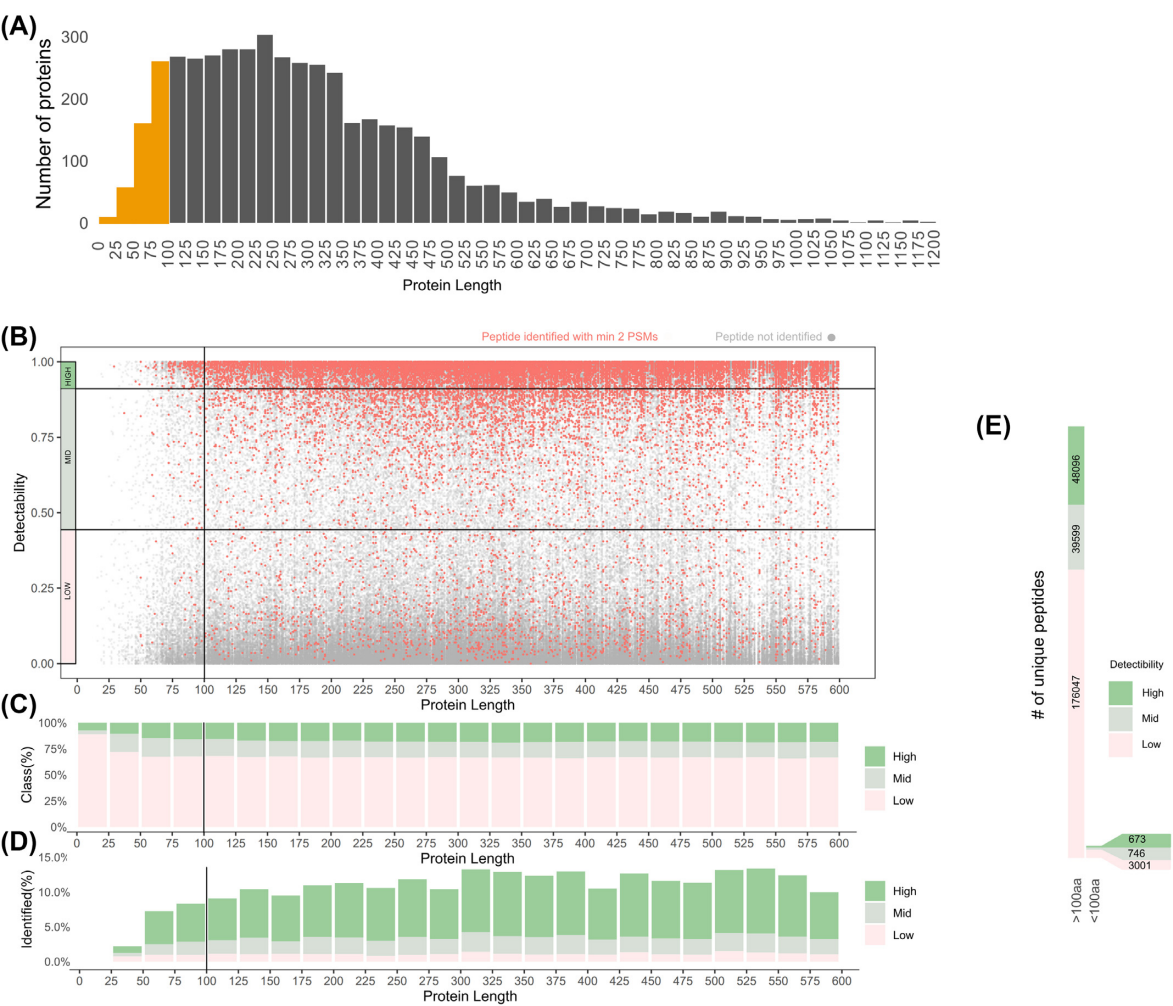
Detectability of peptides produced by *in silico* tryptic digestion of the annotated *S*. Typhimurium proteome. **(A)** Number of *S*. Typhimurium proteins in function of their protein length. **(B)** Distribution of detectability tryptic peptide scores in function of protein length. Three detectability classes are distinguished (high, >0.9; mid, 0.9–0.45; and low, <0.45). Peptides with proteomic support (minimum two PSMs) are indicated in red. **(C)**Composition of peptide detectability classes in function of protein length. **(D)** Peptide detectability classes with proteomic support in function of protein length. **(E)**Numbers of unique peptides in distinct peptide detectability classes for SEPs versus all other proteins.

**Figure 2.**
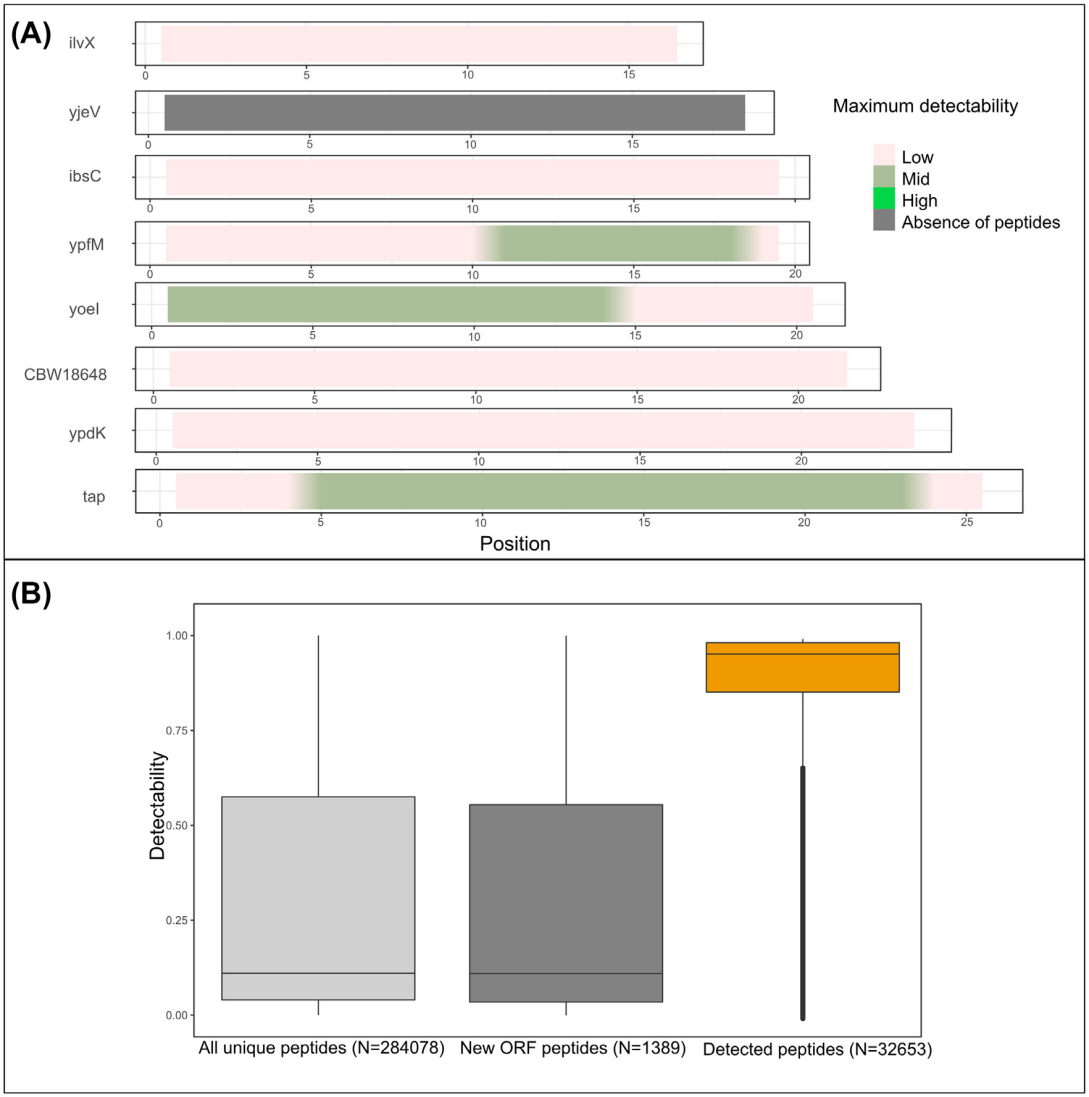
Properties of peptides derived from small proteins. **(A)** Peptide detectability coverage plot of the eight SL1344 annotated SEPs smaller than 25 aa in length, with color representing the detectability score of a peptide. The peptide coverage plots indicate the peptides of the highest detectability at each position. **(B)** Detectability distribution for all unique theoretical tryptic peptides originating from annotated proteins, theoretical unique tryptic peptides originating from novel proteogenomics-detected proteins and unique peptides identified by proteomics in this study.

Rationally, a major reason for missing SEP identifications in proteomics datasets is their short size leading to a limited number of detectable tryptic peptides. Strikingly, while 482 SEPs constitute ∼10% of the annotated *S*. Typhimurium proteome (Fig. 1A and B), they solely produce 4420 peptides (mass range of 600–4600 Da, length of ≥7 aa) or 2.5% of all identifiable peptides. In addition, 21 proteins (of which three SEPs) have no UTPs within the detectable mass range. As evident from Fig. 1A and B, the majority (77%) of database annotated *S*. Typhimurium proteins are between 100–500 aa in length. These proteins also produce the bulk of specific UTPs. Besides being theoretical peptides plausibly generated by trypsin digestion, MS detectability represents an important limitation, i.e. some peptides are refractory toward MS-based detection due to incompatible physicochemical properties such as high hydrophobicity or charge. Such peptide features have been used in machine learning algorithms to predict the detectability of peptides, with a recently developed algorithm being the advanced proteolytic peptide predictor (AP3) (Gao *et al*. 2019). In Fig. 1B, the AP3 predicted peptide detectability (0: low detectability, to 1: high detectability) of theoretical peptides (gray dots) was plotted in function of the length of their respective protein. In addition, peptides identified in this study (at least two PSMs) were indicated in red (Fig. 1B). Globally, out of 32 171 peptides identified, almost 70% are highly detectable (22 734 peptides, detectability > 0.9, dark green class), 20% have medium detectability (6383 peptides, detectability 0.9–0.45, light green) and 10% low detectability (3378 peptides, detectability < 0.45, pink). The proportion of theoretical, highly detectable peptides increases with protein length to reach a plateau of ∼12% when over 150 aa in length (Fig. 1C) and with a significant lower proportions of highly detectable peptides (i.e. 6% on average) originating from proteins smaller than 50 aa in length. This downward trend is particularly evident for SEPs below 25 aa in length (Figs 1C and 2A). Viewing the peptide identification rate, i.e. the proportion of identified theoretical peptides, it is thus indisputably that SEPs suffer from lower peptide identification rates (2.5% for proteins < 50 aa, 4.5% for proteins <100 aa), where the rate remains relatively stable for longer proteins at ∼9% (Fig. 1D). It can be observed that peptide detectability of SEPs represents a strong influencing factor when considering their MS-based detection, with SEPs only producing 673 unique peptides originating from 421 SEPs (i.e. 1.6 unique peptides per SEP on average) with a detectability score higher than 0.9 (Fig. 1E). Overall, identified peptides had a high detectability score median of 0.88, in sharp contrast to the 0.18 median detectability score of all theoretical tryptic peptides of the *S*. Typhimurium proteome (Fig. 2B). In summary, the relatively lower proportion and lower absolute number of MS-detectable peptides in case of SEPs (Fig. 1E) is convincingly a strong influencing factor for their general lower proteomic identification rate.

When focusing on all eight database annotated protein entries smaller than 25 aa (Fig. 2A) it becomes clear that scarcity of detectable peptides—with even no theoretical peptides with detectability score above 0.9—represents a major problem for MS detection of SEPs. This finding is further accentuated by the fact that one such SEP completely lacks identifiable UTPs (CCG27798; yjeV) and four displaying only UTPs with low detectability scores (<0.4). Evidently, the same phenomenon influences also the proteomic detection of potentially unannotated SEPs in proteogenomic efforts as illustrated by the example of a newly identified sORF, sORF 23, lacking any detectable peptides, while nonetheless showing strong Ribo-seq support (Fig. S4, Supporting Information).

### SEP properties contributing to challenging proteomic detection

**Figure 3.**
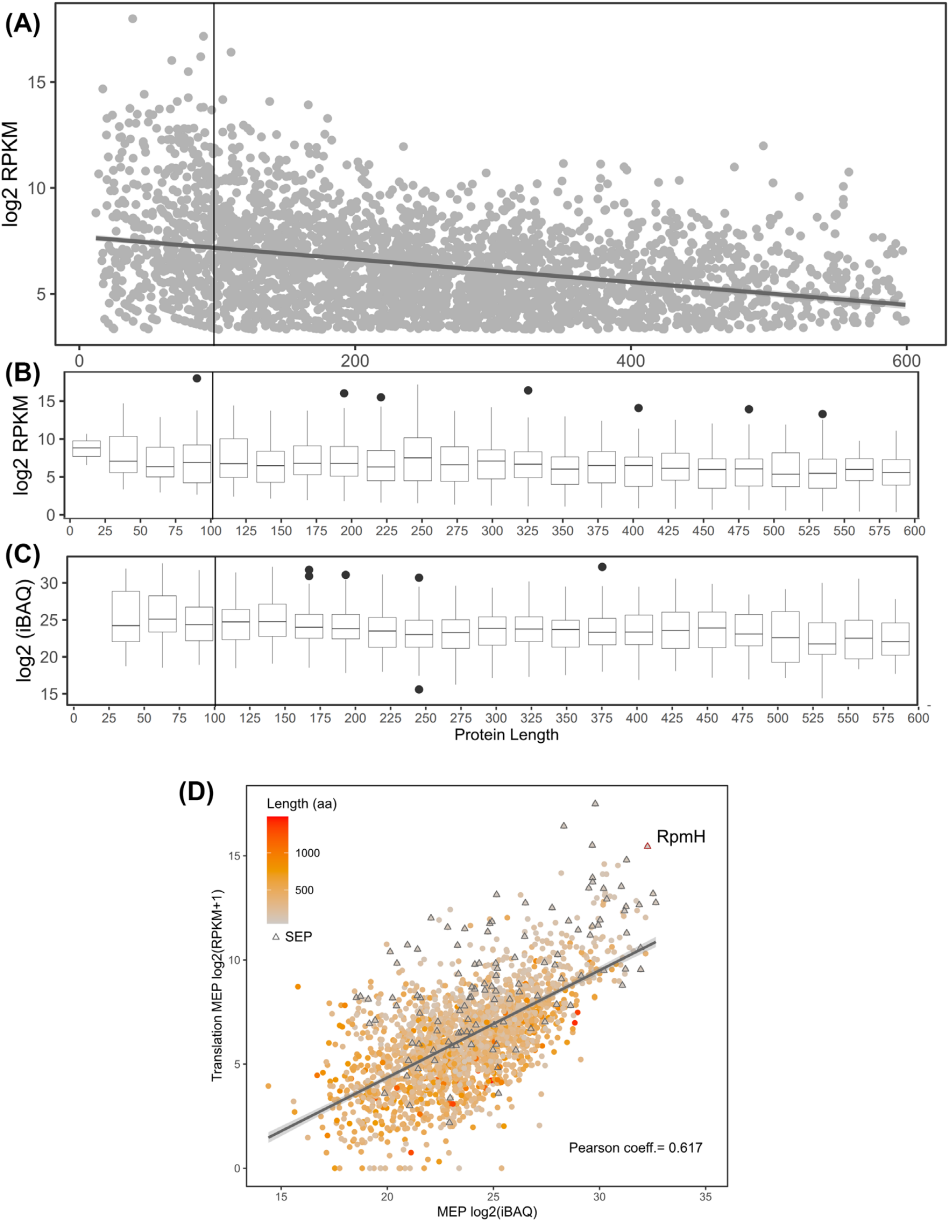
Protein Ribo-seq expression levels in function of protein length. **(A)** Log_2_-transformed RPKM values of all annotated proteins smaller than 600 aa. **(B)** Box plot depicting distribution of translation values per protein length bin. **(C)** Abundance of annotated proteins detected in the proteomics experiments as measured by log_2_(iBAQ) values in function of protein length. **(D)** Correlation between proteomics and translation as measured by ribosome profiling in MEP (OD 0.3 in LB medium). The protein length is marked on a color scale with proteins smaller than 100 aa indicated with triangles. Pearson correlation coefficients are marked in respective graphs. The robustly expressed RpmH SEP described in the text is highlighted. The quantification of RpmH is highlighted as an example of robustly expressed SEP (see manuscript text for details).

The relative low abundance of SEPs has been widely suggested as a confounding factor hindering their proteomics identification. With proteomics being potentially biased against identifying SEPs, we additionally turn to matching translatomics information that is likely unaffected by the same confounding factors. Here, we analyzed this notion by viewing normalized Ribo-seq translation levels of all robustly expressed (RPKM > 5) annotated proteins (Fig. 3A and B). Note, this normalization corrects for ORF length and is here displayed as log_2_-normalized RPKM expression values. Moreover, the typical Ribo-seq read accumulation at the translation initiation and termination sites is corrected for (i.e. signal at 5% of terminal positions was removed from the analysis) as previously described to avoid overestimation of sORF translation levels (Verbruggen *et al*. 2019). It can be observed that SEPs display relatively similar translation levels as compared with longer proteins (Fig. 3B), at least for the expressed annotated proteins considered. This indicates that a generalized lower expression of SEPs is unlikely to constitute a decisive factor accounting for their poor identification rates. In fact, the category of SEPs shorter than 25 aa in length, for which no peptides were identified, displays even slightly elevated translation levels (median log_2_(RPKM) of 8.12) while being affected by poor detectability of their resulting peptides (as illustrated by Figs 2A and 3A and B). With matching proteomics data correlating well with these translatomics findings (Fig. 3C and D), we can conclude that low expression—both among missed annotated proteins and newly detected ones (discussed further in this work)—is thus unlikely to be a main confounding factor specifically hindering the MS-based proteomic detection of SEPs.

**Figure 4.**
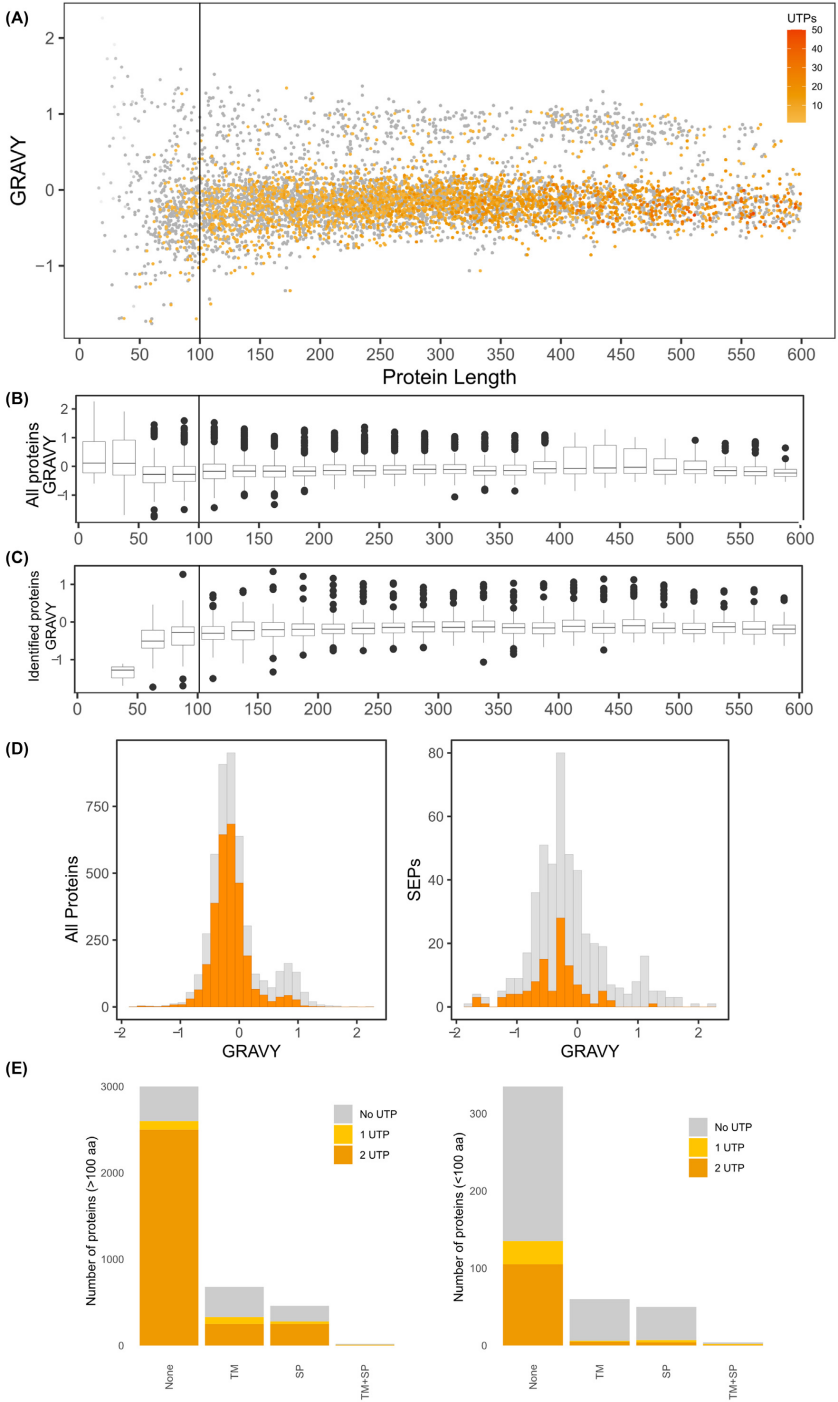
Protein hydrophobicity of the annotated *S*. Typhimurium proteome. **(A)** Distribution of protein hydrophobicity scores in function of protein length. Proteins detected in the proteomics experiments were highlighted in color in function of the number of identified UTPs. **(B)** Box plot depicting distribution of hydrophobicity scores per protein length bin. **(C)** Box plot depicting distribution of hydrophobicity scores of proteomics identified proteins per protein length bin.**(D)**Histogram of hydrophobicity scores of proteins for all annotated proteins (left) and for SEPs (right). **(E)**Domain composition of all proteins (left) and SEPs (right) in the *S*. Typhimurium proteome.

Among other factors postulated to account for the low detectability of SEPs and given that a significant proportion of bacterial SEPs has been predicted to contain (hydrophobic) TM domains in previous studies (Fontaine *et al*. 2011), the hydrophobic nature of SEPs has often been put forward. In this regard, it is striking to note that 9 out of 10 most hydrophobic annotated *S*. Typhimurium proteins are SEPs, and none of them have been identified in our proteomics screen despite good translatomics expression levels (mean log_2_ RPKM of 11.279). To evaluate to what extent hydrophobicity affects SEP detection, we calculated the grand average of hydropathy (GRAVY) metric for all annotated proteins. This gives an indication of protein hydrophobicity/hydrophilicity, with higher GRAVY values indicating more hydrophobic proteins. It can be observed that small proteins exhibit a more extreme range of hydrophobicity scores compared with larger proteins (Fig. 4A–D), especially when considering the higher GRAVY distribution in SEPs smaller than 50 amino acids (Fig. 4B). When considering identified UTPs, a set of hydrophobic proteins (GRAVY ≥ 0.5) can be distinguished with poor MS proteomic evidence for all proteins and especially SEPs (Fig. 4C and D). Taken together, MS detection and GRAVY are negatively correlated, meaning that hydrophobic proteins are generally poorly detectable by MS, a property further accentuated for SEPs given their increased average hydrophobicity. Also the observation that in the category of SEPs smaller than 50 amino acids with a mean GRAVY value of 0.198, the by proteomics identified SEPs displayed a mean GRAVY of −1.36, further indicates that essentially, only hydrophilic SEPs of this size category were identified. Moreover, among the properties used in AP3 predictions, high peptide hydrophobicity is considered an indicator of low detectability, further supporting our findings at the protein level (Gao *et al*. 2019). Noteworthy, however, is that hydrophobic proteins (>0.8 gravy) display a mean log_2_(RPKM) expression value of 6.605, which is significantly lower compared with the 7.63 expression value for less hydrophobic proteins (gravy < 0.8) (*P*-value < 0.05). If the same calculation is made exclusively for SEPs then hydrophobic SEPs (gravy > 0.8) are slightly higher expressed log_2_(RPKM) of 7.42 vs 6.985 for hydrophilic SEPs.

Related to the strong hydrophobicity of SEPs, we investigated their domain structure, namely, the occurrence of signal peptides (SPs) and TM helices in their sequence. Importantly, SPs are easily mistaken as TM helices given their similar biophysical properties (Krogh *et al*. 2001). Therefore, we first predicted SPs after which TM helix prediction was performed on *S*. Typhimurium proteins with predicted SP-containing proteins removed. As the relative numbers of TM helices and SPs predicted show relatively similar proportions in SEPs and longer proteins, the MS identification is drastically affected in case of SEPs (Fig. 4E). For instance, only 7 out of 71 (∼10%) TM helix-containing SEPs were identified (≥2 PSMs) (i.e. CBW19682, CBW17714, CBW19928, CBW20018, CBW17862, CBW18094, CBW20286), whereas 182 out of 404 (45%) TM helix-containing proteins larger than 100 aa were identified (Fig. 4E). This is in stark contrast to 130 out of 323 (∼40%) SEPs and 2530 out of 3022 (∼84%) longer proteins identified without predicted TM domains or SPs. Likely, the greater relative portion of these structural domains present in SEPs signifies another contributing factor hindering their proteomics detection, and again in line with the increased hydrophobicity aspect of such motifs. This is especially apparent when peptide coverage of detected membrane proteins is considered, with TM proteins showing median coverage of 15.68% (with 30% maximal theoretical coverage when considering highly detectable peptides) while soluble proteins display a 43% median coverage (with 54% maximal theoretical coverage when considering highly detectable peptides).

Similarly to high hydrophobicity, potential low stability of SEPs could hinder their MS discovery. In the absence of proteome-wide data of bacterial protein turnover we turned to stability predictions that shown efficacy in reflecting some aspects of (bacterial) protein instability (Guruprasad *et al*. 1990, Gamage *et al*. 2019). To examine this, we calculated the instability index as described by Guruprasad and colleagues (Guruprasad *et al*. 1990). This index predicts protein stability based on the amino acid composition of the protein, namely the bicodon occurrence frequencies, a proxy utilized by many protein stability predictors (Pucci *et al*. 2017, Yang *et al*. 2019, Chen *et al*. 2020). Proteins with an instability index lower than 40 are predicted to be stable, while higher values represent a good indication of protein instability. Similar to GRAVY scores, we observe that SEPs display a more extreme variation in instability indexes, with SEPs smaller than 25 aa displaying clearly elevated instability indexes (Fig. S5A and B, Supporting Information) while larger SEPs do not deviate from the index distribution observed for longer proteins. However, intriguingly, when only considering identified proteins (Fig. S5C and D, Supporting Information), we observe that identified proteins smaller than 50 aa in size display relatively elevated instability indexes pointing toward robust identification of, at least some SEPs predicted as being (relatively) unstable. Despite this subtle trend, the methodology used cannot predict if proteins might undergo (targeted) proteolysis *in vivo* (Gamage *et al*. 2019), so instability of at least some SEPs cannot be excluded as clearly demonstrated by recently observed improved validation rate of novel bacterial SEPs in the presence of Bortezomib, due to lowered protein degradation rates (Stringer *et al*. 2021).

Next to Ribo-seq expression levels, we viewed protein abundance (iBAQ) measured by MaxQuant for 3053 proteins identified by proteomics (≥1 UTP, min two PSMs; Fig. 3D and E). It can be observed for all proteins and SEPs, that Ribo-seq RPKM and MS-based protein iBAQ values are positively correlated (mean Pearson coefficient of 0.67). Moreover, SEPs with sizes as small as 46 residues, e.g. RpmH, can be among the most abundant proteins expressed, with this particular protein being the third and fourth most abundant protein in our proteomics and Ribo-seq datasets, respectively (highlighted in Fig. 3D). Admittedly, small ribosomal proteins are a special case of robustly expressed SEPs, but their removal from the analysis did not reveal a significant change in the average expression levels between SEPs and longer proteins (chi square test). By and large, proteomics detected SEPs display robust expression throughout investigated growth conditions (Table S1, Supporting Information).

### Low numbers and poor detectability of tryptic peptides, but not general abundance and predicted stability, are major limiting factors for missing SEP identifications

Compounding effects of the various properties described earlier (protein hydrophobicity, peptide detectability etc.) all contribute to the observed underrepresentation of SEPs in proteomic datasets. To estimate the overall probability of SEP detection, a multivariate analysis was performed using logistic regression for dichotomous outcomes (adjusted odds ratio) as described in detail in Lee *et al*. (2009) and Grant *et al*. (2019). The detection probability of a SEP-derived peptide was calculated from a multivariate, compounding statistical model. Factors included in the multivariate analysis are Ribo-seq expression levels (RPKM values), number and detectability scores of tryptic peptides originating from the SEP, hydrophobicity of the protein and its instability as measured by instability index. Using this model to estimate detection chances of individual peptides the adjusted odds ratio for SEP detection could be calculated. Given the partial stochastic aspect of DDA-based peptide identification in a given MS experiment, the probability of SEP identification equals 0.0342. This value closely resembles the contribution of SEP-originating peptides in the pool of all tryptic peptides (∼2.5%) hinting toward a highly complete description of experimentally observed variance. Based on this analysis, we can delineate that scarcity and poor detectability of tryptic peptides originating from SEPs jointly explain over 75% of variability observed in SEP detection and thus represent the dominant limiting factors for their MS-based detection. This finding offers a more nuanced view of the factors limiting SEP detection compared with low abundance and stability generally put forward as major causes of this phenomenon.

### Riboproteogenomic discovery of unannotated SEPs in *Salmonella*

**Figure 5.**
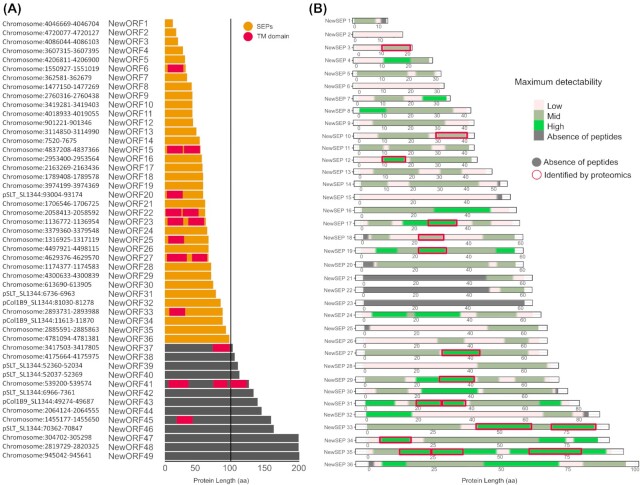
Riboproteogenomics reannotation of the *S*. Typhimurium genome. **(A)** Chromosomal position, length and domain composition of newly Ribo-seq called intergenic or (partially) overlapping ORFs. **(B)** Peptide detectability coverage plots for Ribo-seq predicted SEPs with the proteomics identified peptides highlighted in red. Detectability of peptides is coded on a color scale.

**Figure 6.**
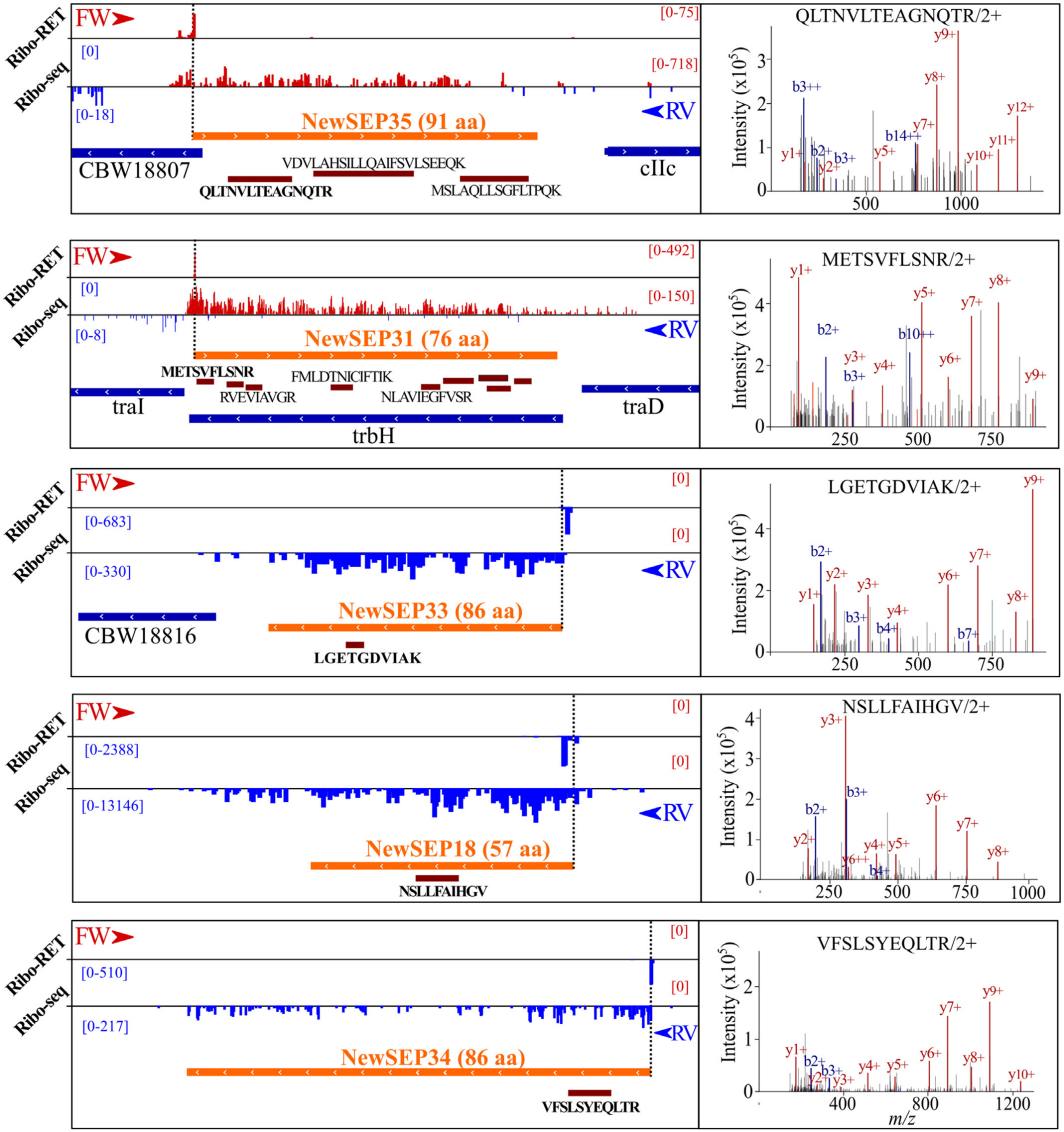
Newly discovered SEPs with supporting proteomics evidence. Novel SEPs are presented with matching examples of assigned peptide fragmentation spectra.

**Figure 7.**
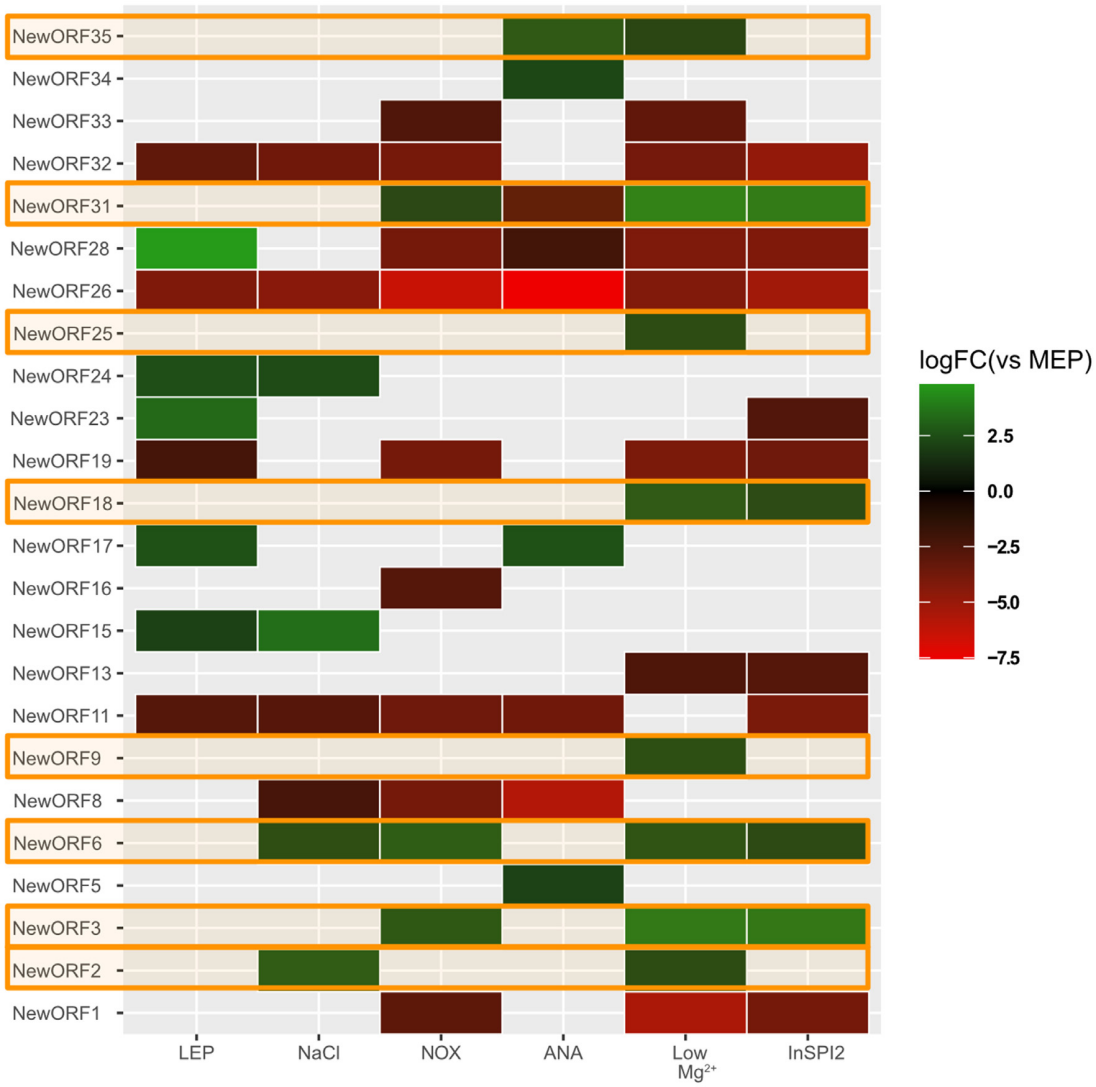
Expression regulation of unannotated intergenic sORFs at the level of translation (measured by Ribo-seq). Condition-specific expression of novel intergenic ORFs significantly differentially expressed between the MEP (OD 0.3 in LB medium) condition and other growth conditions investigated. Only ANOVA significant regulation is presented with log_2_ fold change in color scale. Novel sORFs regulated in SPI2-inducing conditions have been highlighted in orange.

As ORF size impacts the accuracy of genome annotation algorithms, sORFs and their corresponding SEPs are in particular affected by underannotation (Baek *et al*. 2017). In order to deepen our understanding of the *S*. Typhimurium genome, we inspected the Ribo-seq and Ribo-RET datasets for pervasive translation outside of annotated genes. Using the datasets obtained, we applied our Ribo-seq based gene detection search for novel genomic elements (Willems *et al*. 2020). In total, 243 unannotated translation products were detected. Next to small proteins, and in line with our previous findings (Ndah *et al*. 2017, Willems *et al*. 2020), we detected a number of errors persisting in current genome annotation of *S*. Typhimurium. To this end, protein truncations (115) and extensions (79) of annotated genes have been found indicating incorrectly assigned start sites. Fifty-four of such novel genomic elements have been matched by two or more UTPs in our proteogenomic pipeline search highlighting the need for continuous data-driven reannotation efforts. As such reannotation efforts have previously been described by us and others (Giess *et al*. 2017, Ndah *et al*. 2017, Willems *et al*. 2020), in this work we focused on sORFs discovery and their resulting protein products—SEPs. Among the 49 newly discovered intergenic ORFs, 36 encode proteins smaller than 100 aa (Fig. 5; cross-referenced with previous studies as indicated in Table S2, Supporting Information). The newly identified sORFs show robust expression throughout the investigated growth conditions (average log_2_(RPKM) of 9.68 vs 8.64 for all proteins; average log_2_(IBAQ) of 24.16 vs 23.32 for all proteins). Using matching proteomics data, our recently published proteogenomic pipeline making use of six-frame translation (Willems *et al*. 2020) identified 433 (251 with at least two PSMs) new peptides originating from database unannotated genomic locations (Table S2, Supporting Information). In total, such newly discovered peptides have been matched to 321 either novel, incorrectly annotated or unannotated genomic elements. When considering SEP identification, 12 of the 36 (33%) novel sORFs predicted by Ribo-seq have been confirmed by at least one unique peptide (≥2 PSMs) displaying good quality fragmentation spectra supporting their confident identification (Figs 5B and 6). Eight out of the 36 (22%) newly discovered sORFs are predicted to encode SEPs with TM domains (Fig. 5A), slightly more than in case of annotated SEPs (15%). Among the 36 newly discovered sORFs, 25 display ANOVA significant (*P*-value < 0.05) Ribo-seq expression levels when considering late exponential growth versus the five other growth conditions tested by Ribo-seq, further indicating their potential biological role (Fig. 7). Notably, eight novel sORFs (new sORF 2, 3, 6, 9, 18, 25, 31, 35) were significantly upregulated in *Salmonella* pathogenicity island 2-inducing growth conditions (PCN and PCN low Mg^2+^) that mimic the intra-vacuolar growth conditions of infecting *S*. Typhimurium (Srikumar *et al*. 2015) (Fig. 7, highlighted in orange; average log_2_ FC over MEP of 2.21). Ribo-seq signal of a representative example (new sORF 31) is shown in Fig. S6 (Supporting Information).

In order to further evaluate potential biological functions of novel sORFs detected in this study, we performed homology analysis using protein BLAST for the putative SEP sequences against the bacterial RefSeq protein database. This revealed that all but three (new sORF 1, 5 and 13) of the SEPs detected by our approach share, at least partial, sequence homology to proteins annotated in other bacterial species. Half of such SEPs (16) were hypothetical and uncharacterized proteins present in either other *Salmonella* strains or other members of the Enterobacteriaceae family. Such hypothetical, multispecies bacterial protein records in the NCBI RefSeq database (WP entries) were introduced recently to avoid redundancy and are managed independently from the genome accessions (Tatusova *et al*. 2016). One example of this is the new sORF 34 that matches the multispecies hypothetical protein WP_000343102.1 annotated in Enterobacteriaceae. Similarly, new sORF 35 matches a truncated version of hypothetical protein WP_204903213 annotated in *Salmonella enterica*. On the other hand, certain homology matches can provide valuable insight into the biological function of newly detected SEPs. New sORF 26 matches the VapB antitoxin of the type II toxin-antitoxin system of *Salmonella enterica* (Winther and Gerdes 2011). This protein has thus far not been annotated in SL1344 strain used in this study and next to existing homologs, strong genomic and proteomics support its true expression again showcasing the validity of our proteogenomics approach. Similarly, the 17 amino acid long SEP encoded by new sORF 2 (MDPEPTPLPRWRIFLFR) showed full homology with the *mgtL* encoded regulatory leader peptide of the magnesium-transporting ATPase MgtA, serving a molecular proline level detector function (by its four encoded prolines) and regulating osmotic shock induction (Park *et al*. 2010). Regulatory SEPs represent a distinct category of SEPs that intriguingly may display highly deviating amino acid and dipeptide compositions. Other functional indications of newly discovered SEPs include involvement in ribosomal structure (50S ribosomal protein L36 homolog; new sORF 4), magnesium transport (MgtS homolog; new sORF 6, SlyB homolog; new sORF 8), lipoprotein processing (patatin homolog; new sORF 15) and general bacterial metabolism (PTPS synthase homolog; new sORF 13).

## Discussion

The elusive nature of SEPs in proteomics has long been explained by often incompletely studied assumptions pointing toward their low abundance, unstable nature or broadly undefined incompatibility with MS-based detection. In this study we carefully examine such inherent properties of SEPs and point toward a combination of factors that jointly explain the challenging proteomic detection of SEPs. Logically, low number of unique tryptic peptides produced by SEPs represents the biggest challenge in this process (explaining up to 60% of variance observed). The scarcity of tryptic peptides produced points directly to possible improvements that could be achieved by generating proteomics datasets using alternative proteases (Tran *et al*. 2011). For instance, *in silico* chymotrypsin digestion results in ∼30% more theoretically identifiable peptides from annotated *S*. Typhimurium SEPs (considering two missed cleavages and high specificity as defined by ExPaSy; https://web.expasy.org/peptide_cutter/peptidecutter_enzymes.html). Despite this, trypsin is often preferred due to its enhanced specificity, consistency in cleavage site selection and resulting C-terminally charged peptides displaying improved ionization in MS experiments (Giansanti *et al*. 2016). In *S*. Typhimurium, merely 2.5% of all tryptic peptides (falling within MS detection size limits) are produced from proteins shorter than 100 aa in size. This is further exacerbated by the low detectability scores of the majority of such peptides as strikingly ∼70% of these have detectability scores lower than 0.45 while the average detectability of proteomics-identified peptides in this study was 0.856 (Fig. 1; Fig. 2B). Thus, on average, only one in eight tryptic peptides originating from the *S*. Typhimurium proteome displays high MS detectability. This fact, further compounded by the hydrophobic nature of many SEPs and their frequently predicted membrane localization, nearly completely explains the challenge posed for the MS-based discovery of this understudied class of proteins. Importantly, low abundance and predicted lower stability of SEPs often postulated in literature were poorly supported as important contributors to subpar detection of SEPs in our analysis. In fact, SEPs identified in this study displayed relatively high instability measures as predicted (Fig. S5, Supporting Information) and did not deviate significantly from the overall abundance distribution as inferred from Ribo-seq. Relatively good validation rates for some of the other landmark studies (Baek *et al*. 2017, Venturini *et al*. 2020) potentially support the observation that stability might not be a limiting contributor to the subpar proteomic SEP detection. However, computational descriptors of stability, such as the instability index utilized in this study display significant flaws as they for example fail to capture (specific) cellular proteolysis events and rather reflect thermal fold stability of a protein. Moreover, recent findings indicated that inhibition of protease ClpP led to improved SEP validation in *E. coli* suggesting that rapid degradation might affect a significant fraction of SEPs (Stringer *et al*. 2021). However, prior to generalizing the concept of ClpB inhibition-mediated stabilization of SEPs, a proteome-wide assessment on protein turnover (and stabilization following ClpP inhibition) is deemed required before generalization of possible differences in SEP versus non-SEP stabilities.

Inherent to their small protein size, a substantial fraction of SEPs found in proteomic datasets are only identified by a single UTP (19% vs 8% for longer proteins). This is problematic as detection of more peptides often serves as a guideline for trustworthy protein identification and quantification, making it nearly impossible to identify and quantify SEPs in a comprehensive manner. From available MS evidence of SEPs in *Mycobacterium pneumoniae*, it was shown that the category of SEPs with at least two distinct peptides managed to distinguish true positives, whereas false positives more frequently remained supported by one peptide hit (Miravet‐Verde *et al*. 2019). However, as we demonstrate in the current and a previous proteogenomic study (Willems *et al*. 2020), supporting such identifications with one or more lines of orthogonal riboproteogenomic evidence, besides spectral quality features including the comparison to predicted fragment ion intensities, can greatly improve our confidence in the true nature of sORFs and SEPs. Transcriptomics, translatomics and translation initiation delineation all can provide direct supporting evidence of protein production. With all eight annotated *S*. Typhimurium proteins smaller than 25 aa only producing three peptides of medium detectability score (Fig. 2A), such orthogonal data might often be lacking or provide the sole evidence of very few endogenously expressed SEPs. Previously, to gain further support for such one-hit wonders and to incorporate quality of spectral matches into the equation, synthetic peptides were synthesized and resulting MS^2^ spectra compared (Friedman *et al*. 2017). It should be noted that by comparing to MS^2^PIP-predicted spectra (Degroeve and Martens 2013, Degroeve *et al*. 2015), a somewhat similar rationale is incorporated into our proteogenomic pipeline utilized in this study (Willems *et al*. 2020) (Fig. S7, Supporting Information). MS^2^PIP predictive models are however trained on tryptic data and perform less optimal for peptides not ending on Arg or Lys at their C-terminus (Wilhelm *et al*. 2021). However, with another spectral predictor—PROSIT (Gessulat *et al*. 2019)—recently including predictions of non-tryptic MHC class peptides, dynamic developments in the field of computational proteomics hold the promise of rapid improvements in this area (Wilhelm *et al*. 2021).

Despite the growing number of valuable efforts to identify and catalog small bacterial proteins, the proteomic evidence of such translation events is often scarce for reasons demonstrated by us in this study (Venturini *et al*. 2020). As high quality omics datasets become available in public repositories for a growing number of species (Van Opijnen and Camilli 2012, Baek *et al*. 2017, Ndah *et al*. 2017, Vanorsdel *et al*. 2018), the metaanalyses concatenating and cross-referencing the findings will be in high demand (Nielsen and Krogh 2005, Venturini *et al*. 2020). With over 3000 confidently identified and quantified proteins (∼65% of the proteome and 74% of the expressed proteome; 5 RPKM cutoff) we provide a comprehensive compendium of the expressed *S*. Typhimurium proteome. The strength of the integrative approach used in this study relies on multiple factors. As previously clearly demonstrated in case of *E. coli* proteome (Schmidt *et al*. 2016), the use of complementary growth conditions aids in increasing proteome coverage. For instance, when comparing protein identifications obtained from MEP with SPI2-inducing low pH minimal media (PCN), 128 and 218 proteins are uniquely identified in the two conditions, respectively. Moreover, matching genomic (Ribo-seq and Ribo-RET) datasets were generated to support such condition specific identifications. The utilization of differential growth conditions can also point toward the functionality of newly discovered SEPs. Previous transcriptomic studies pointed to infection-like expression patterns observed in SPI2-inducing conditions such as the (low Mg^2+^) SPI2-inducing conditions utilized in our study. These conditions correlated highly with expression program displayed by the intravacuolar *S*. Typhimurium population upon infection (Srikumar *et al*. 2015). With eight novel SEPs following a similar expression pattern indicative of their relevance in bacterial infection, the potential biological relevance of genome reannotation efforts is demonstrably clear. The comprehensive coverage of both the translatome and the proteome obtained in this study highlights the utility of conditional interrogation of bacterial genomes. Despite major advances in the technology allowing for effective gene detection based on translation data, further improvements in the bioinformatic toolkit are needed, as also acknowledged in a recent benchmarking study comparing sensitivities of bacterial sORF detection (Gelhausen *et al*. 2022).

A multitude of recent efforts have been undertaken to optimize proteomics detection of SEPs. Use of enrichment strategies, alternative proteases and custom search databases have all shown good promise in increasing the identification rates of SEPs (Bartel *et al*. 2020, Fijalkowski *et al*. 2021, Kaulich *et al*. 2021). In one recent example, using aliphatic polymers to enrich for small proteins has shown promise in enhancing the proteomic detection of SEPs (Fijalkowski *et al*. 2021). However, with certain classes of proteins—most notably hydrophobic and TM proteins discussed here—posing particular challenge to proteomics detection, targeted approaches might be necessary for their effective identification (Omasits *et al*. 2017). Further, the gain of using multiple, complementary proteases can further be guided by theoretical analysis and thus benefit from the development of dedicated proteomic detectability predictors, such as AP3 used in this study for tryptic peptide detectability. Using such predictors, we obtain a rational prediction of the depth to which proteomics analyses can feasibly produce supporting evidence for novel SEPs and help to guide targeted efforts based on genomic information. Given the scarcity of possible proteolytic sites in short proteins and the challenge in limited specificity of some proteases, novel digestion protocols proposed need careful consideration (Kaulich *et al*. 2021). Similarly, albeit very useful for identification and validation purposes, and previously demonstrated for *Bartonella henselae* where a complete membrane proteome has been identified (Omasits *et al*. 2017), enrichment strategies mostly forfeit the quantitative aspect of proteomics, making it challenging to apply them in functional studies aimed at investigating protein regulation (Muratovic *et al*. 2015) or when comparing different types of omics data (Willems *et al*. 2020). Importantly, in case of *S*. Typhimurium, the maximal theoretical coverage of the annotated membrane versus soluble proteome was found to be significantly (*P*-value < 0.01) lower, with, respectively, 30% and 54% coverage when considering highly detectable peptides. Furthermore, translatomics data indicate lower expression means of the membrane versus soluble proteome with average log_2_(RPKM) values of 4.98 and 6.02 (*P*-value < 0.01), respectively (Fig. S8, Supporting Information). As such, detectability and expression critically contribute to the membrane protein bias observed in proteomics datasets and may vary (widely) between organisms. Alternatively, continuous developments in topdown proteomics can potentially improve the detection of intact small proteins as recently demonstrated in complex murine brain tissue samples (Davis *et al*. 2018).

Riboproteogenomics applied in this study aided to elucidate features obstructing proteomic identification of SEPs and explaining their missing annotation in bacterial genomes. Next to providing orthogonal evidence for protein expression, genomic techniques can facilitate the creation of custom databases used for proteomics searches (Ndah *et al*. 2017, Verbruggen *et al*. 2019, Bartel *et al*. 2020). With a potent combination of genomic information, novel proteins can be delineated from translation and transcription datasets. Albeit labor intensive, such efforts are adaptable and could readily be applied to other bacterial species where supporting datasets are publicly available. Our recent proteogenomic pipeline demonstrates an adaptable method of achieving such a goal (Willems *et al*. 2020), and previous efforts provided excellent foundations for utilization of public proteomics datasets (Venter *et al*. 2011, Bonissone *et al*. 2013). Popularization of ribosome profiling technique holds the potential of greatly enhancing these efforts, yet is labor intensive and requires optimizations when applied to a new model. Importantly, data analysis protocols lack standardization and universal, easily adaptable pipelines are in high demand (Liu *et al*. 2020).

Discoveries in bacterial biology rely on available and reliable genome annotations. With missing annotation of small proteins, we are missing out on crucial puzzle pieces needed to obtain a comprehensive understanding of bacterial systems. These findings become all more important in case of pathogenic bacteria, where growing danger of antibiotic resistance warrants urgent efforts to fully elucidate the translational landscape underlying the pathogenicity mechanisms employed by the bacterium to establish an infection (https://www.biorxiv.org/content/biorxiv/early/2019/06/10/665208.full.pdf). Large-scale (ribo)proteogenomic efforts therefore hold the key to fully appreciate the complexity of bacterial genomes. Affordable sequencing prices and available software, besides accessibility of publicly available genomic and proteomic resources, will propel the field to deepen our understanding of bacterial systems biology.

## Funding

This work was supported by the European Research Council(ERC) under the European Union's Horizon 2020 research and innovation program (PROPHECY grant agreement no. 803972 to PVD) and by the Research Foundation Flanders (FWO-Vlaanderen) (project number G051120N to PVD). IF was supported by a personal postdoctoral fellowship from FWO-Vlaanderen (grant number 12I5517N).

## Data availability

The proteomics data underlying this article are available in PRIDE database at https://www.ebi.ac.uk/pride/, and can be accessed with PXD029391 identifier. The genomics and metadata underlying this article are available in Open Science Framework (OSF) database at https://osf.io/, and can be accessed with 10.17605/OSF.IO/H3VXZ identifier.

The proteomics data presented in the study are deposited in the PRIDE repository, accession number PXD029391. Ribo-seq datasets, Ribo-RET datasets and associated metadata (Ndah *et al*. 2017) are available through Open Science Framework (OSF) (https://osf.io/h3vxz/?view_only=8e69e3e04f2d43119595237436b42389; DOI: 10.17605/OSF.IO/H3VXZ).

## Supplementary Material

uqac005_Supplemental_Files
