# Supplementary figures and images for: Hidden in plain sight: challenges in proteomics detection of small ORF-encoded polypeptides
Source: Microlife. 2022 May 14;3:uqac005. doi: 10.1093/femsml/uqac005 (PMC10117744; doi:10.1093/femsml/uqac005)

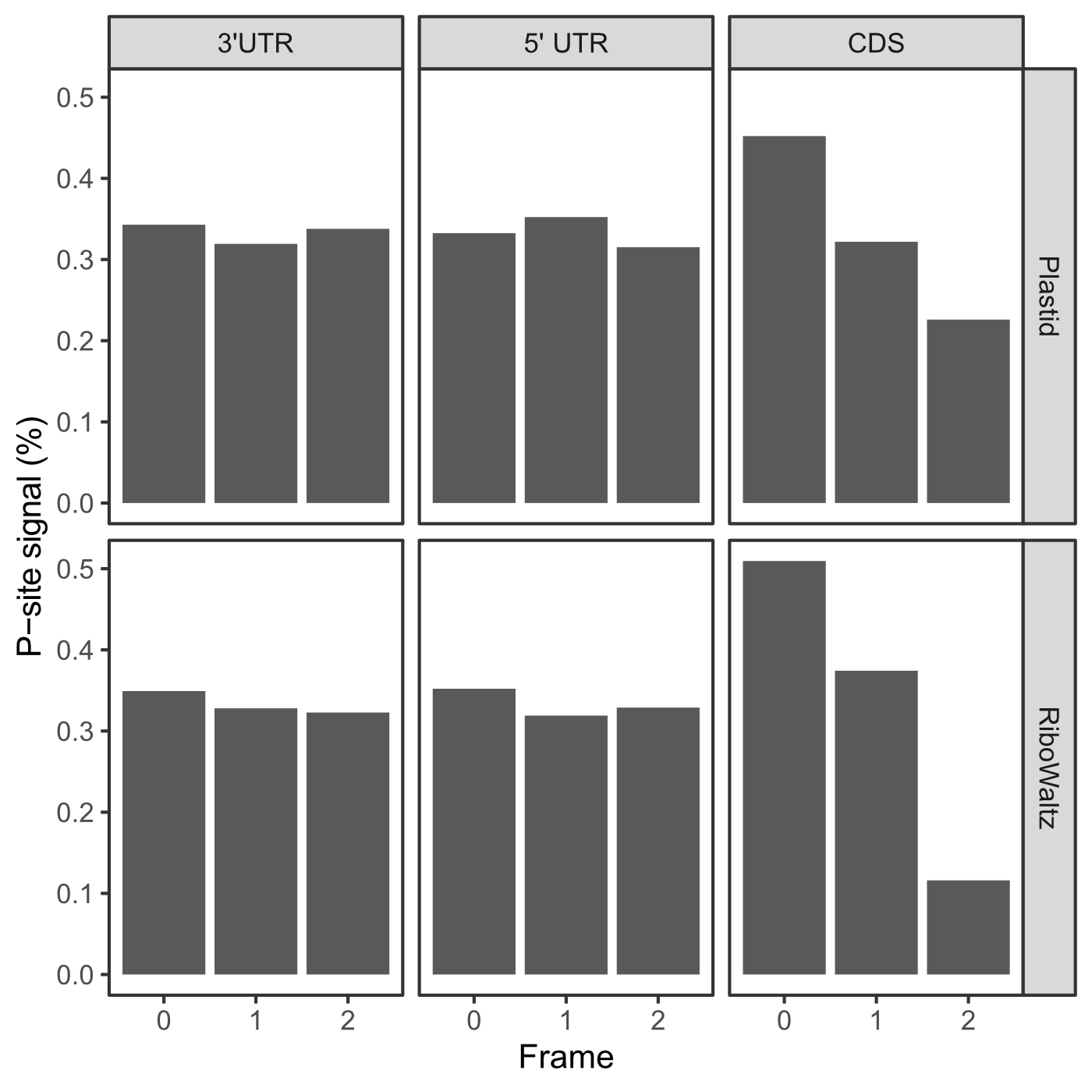

Supplement: uqac005_Supplemental_Files [file uqac005_supplemental_files.zip › SuppFig1.tiff]

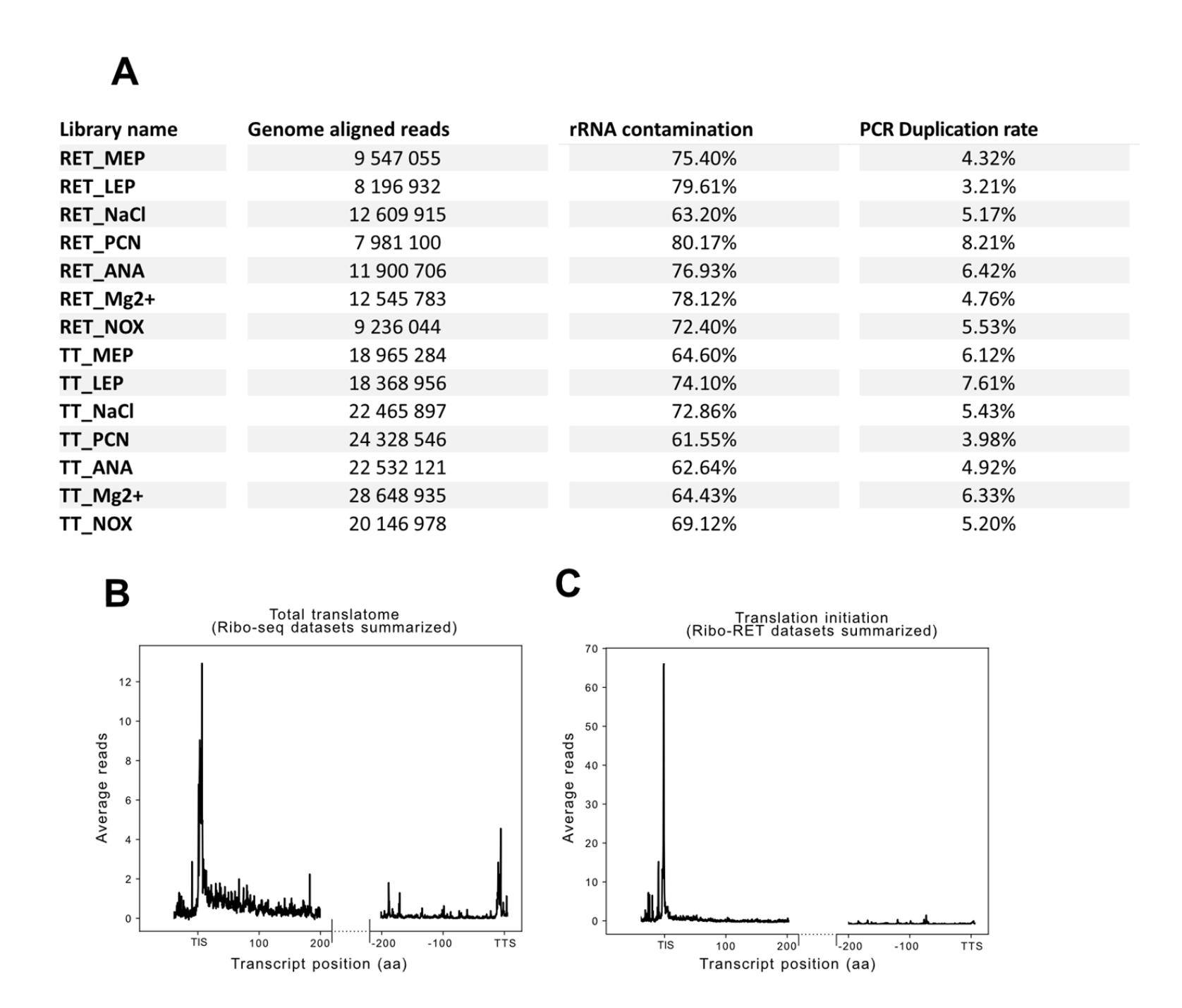

Supplement: uqac005_Supplemental_Files [file uqac005_supplemental_files.zip › SuppFig2.tiff]

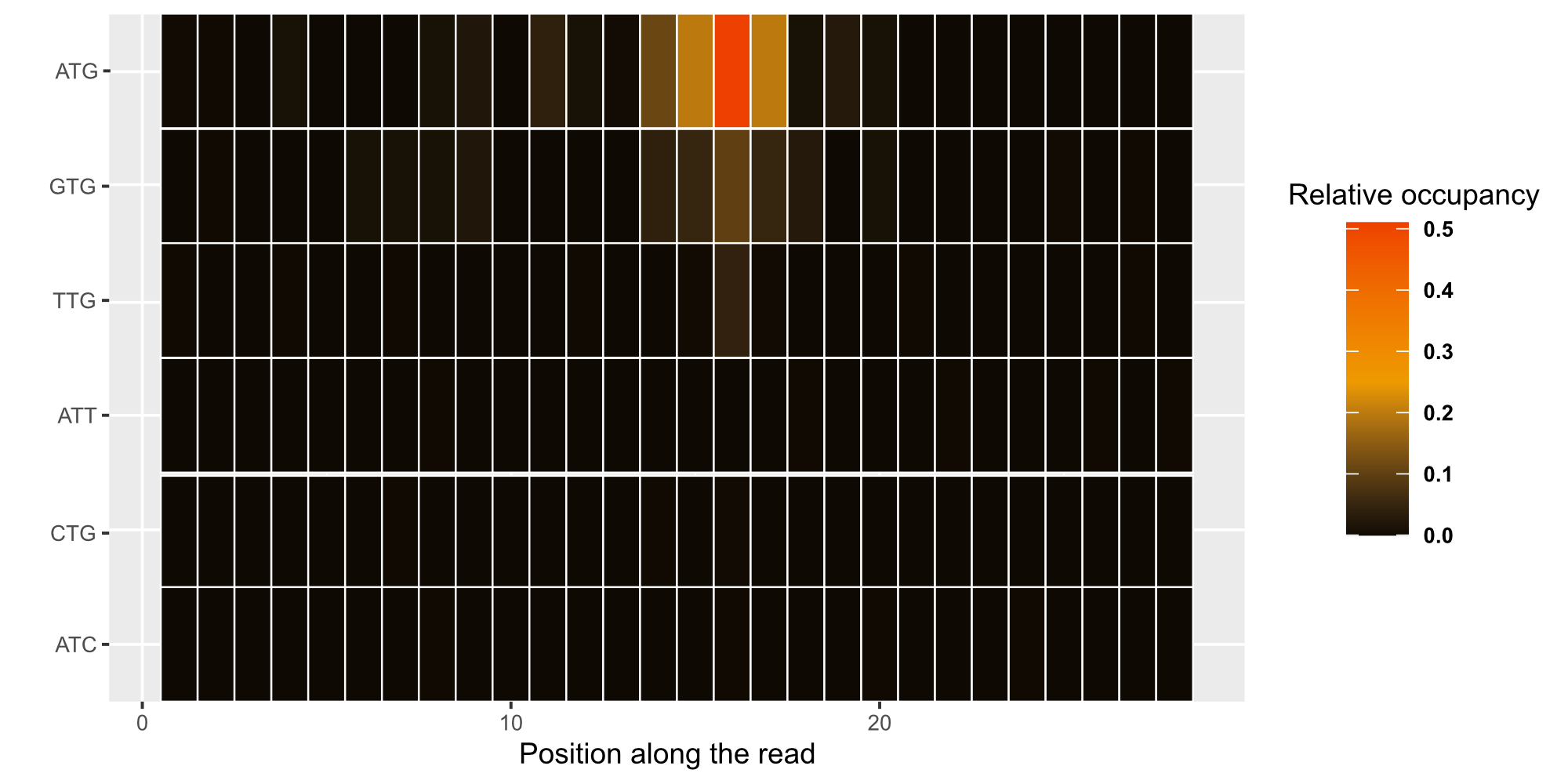

Supplement: uqac005_Supplemental_Files [file uqac005_supplemental_files.zip › SuppFig3.tiff]

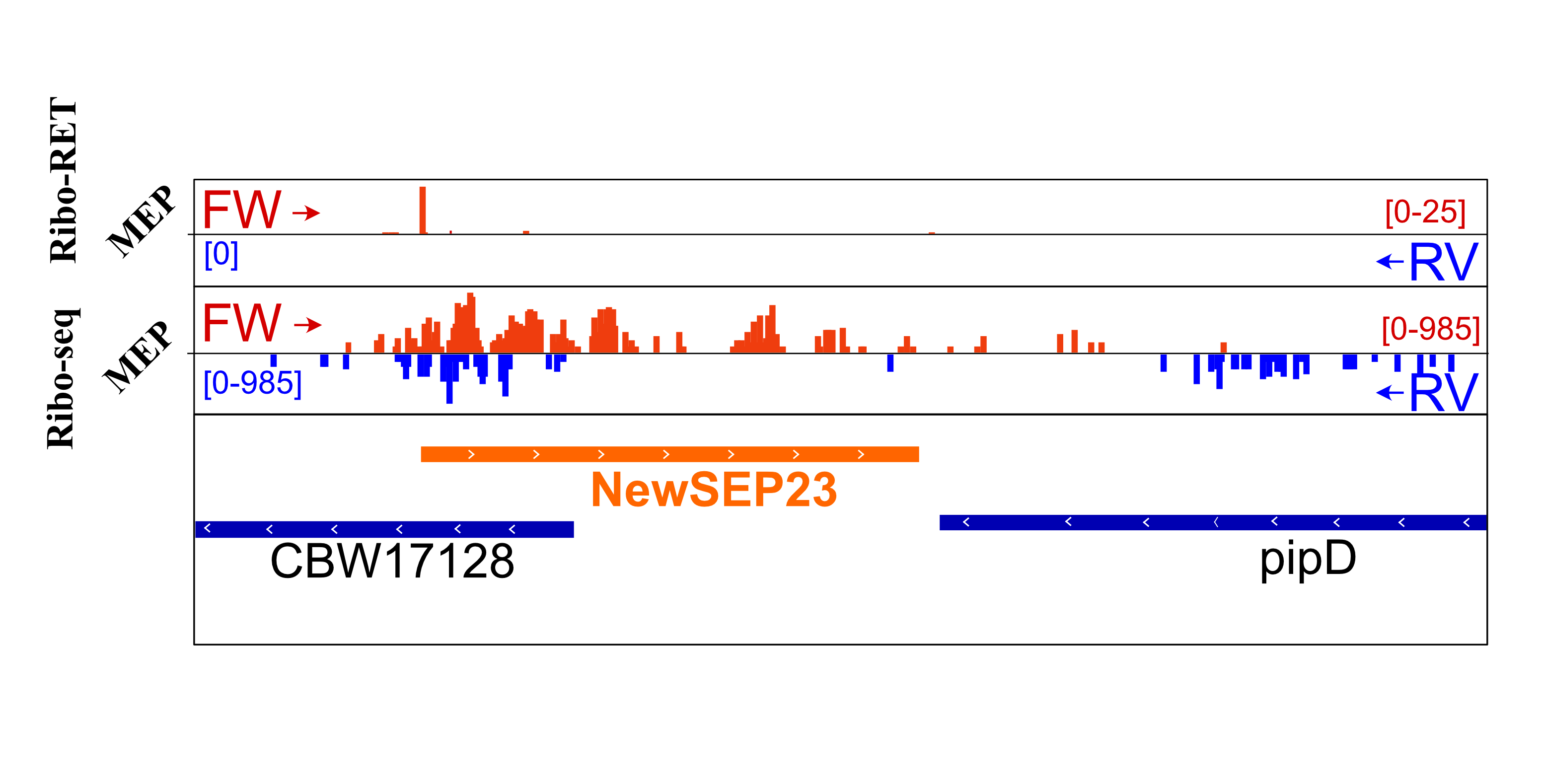

Supplement: uqac005_Supplemental_Files [file uqac005_supplemental_files.zip › SuppFig4.tiff]

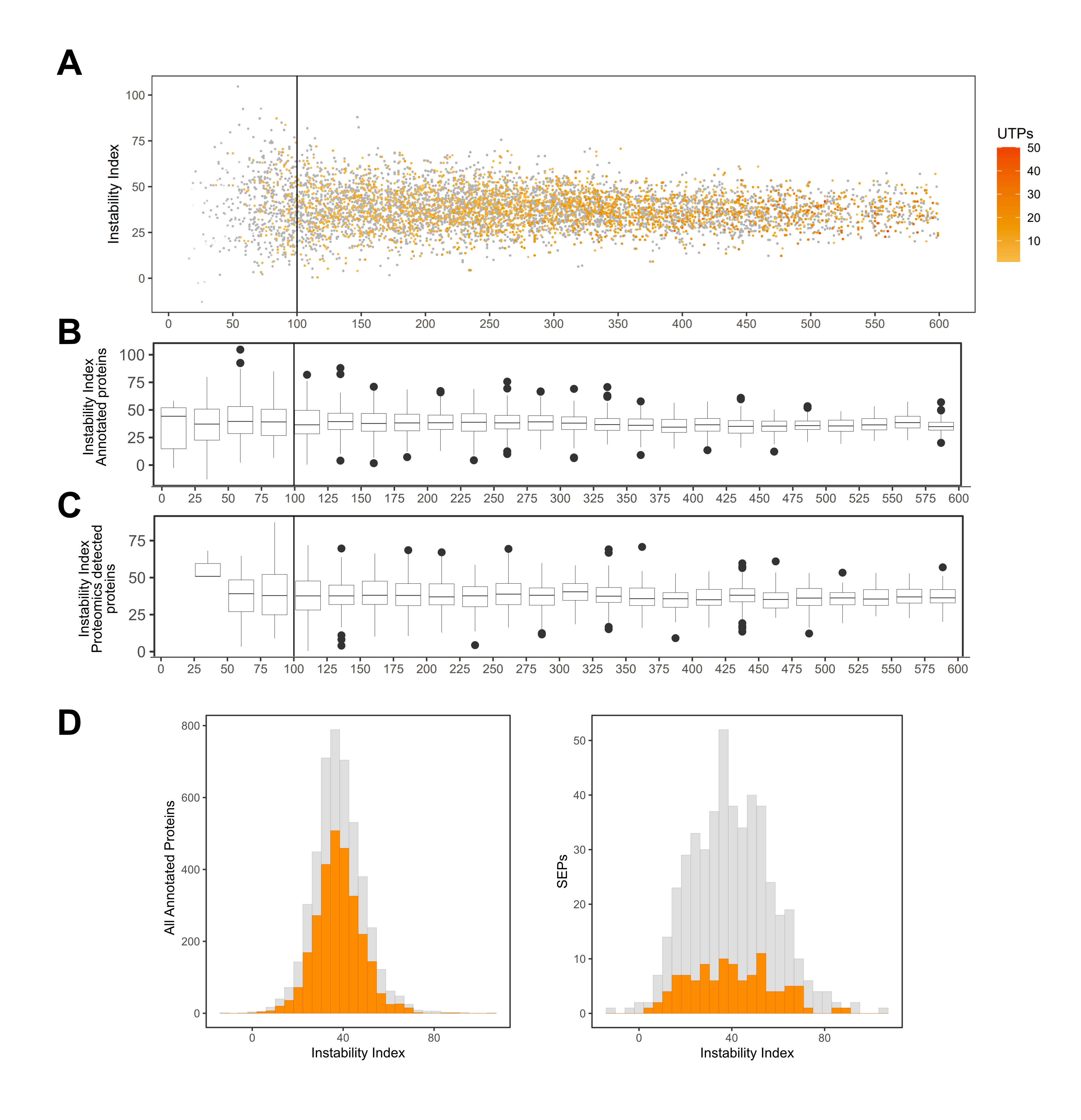

Supplement: uqac005_Supplemental_Files [file uqac005_supplemental_files.zip › SuppFig5.tiff]

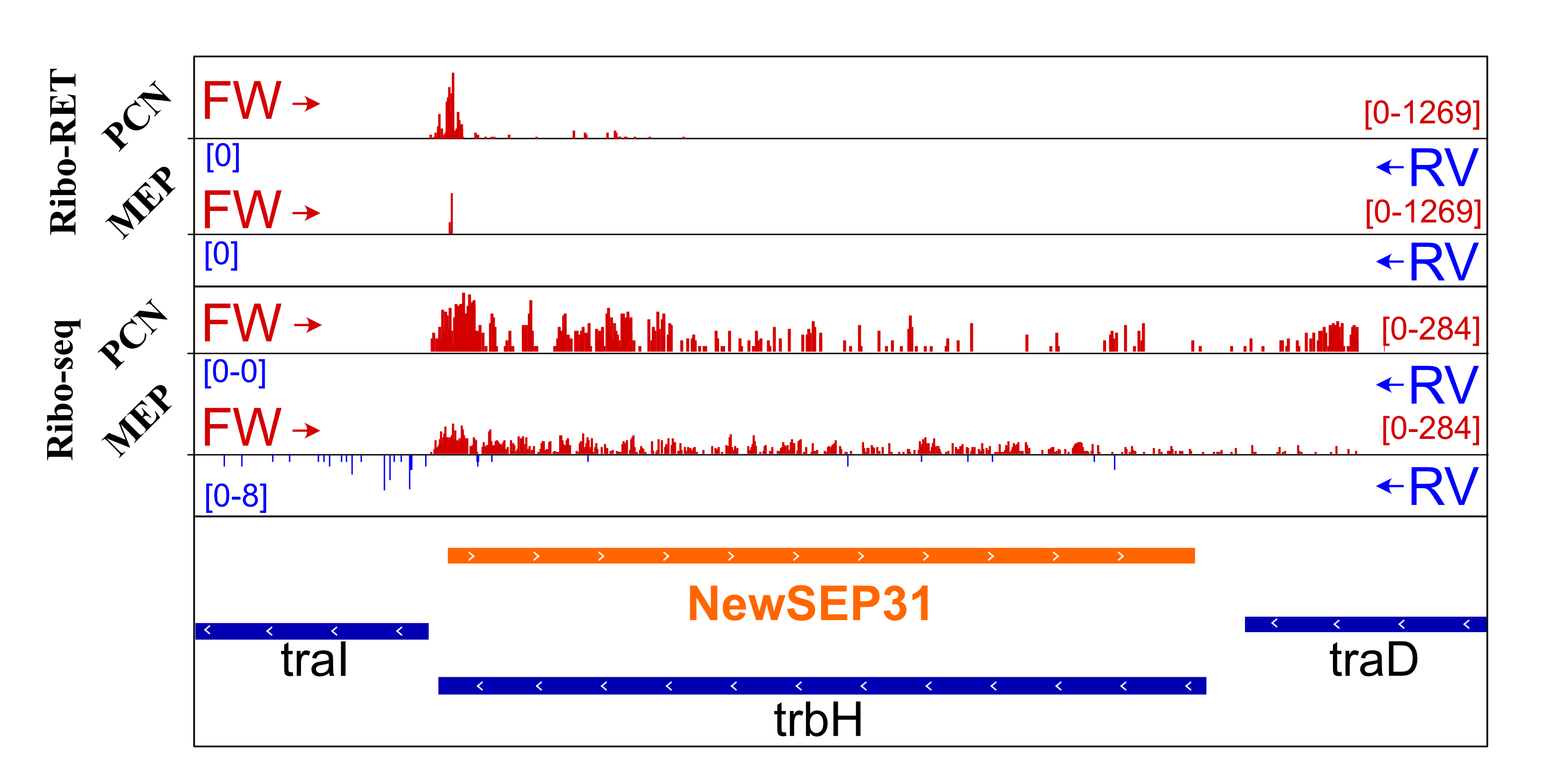

Supplement: uqac005_Supplemental_Files [file uqac005_supplemental_files.zip › SuppFig6.tiff]

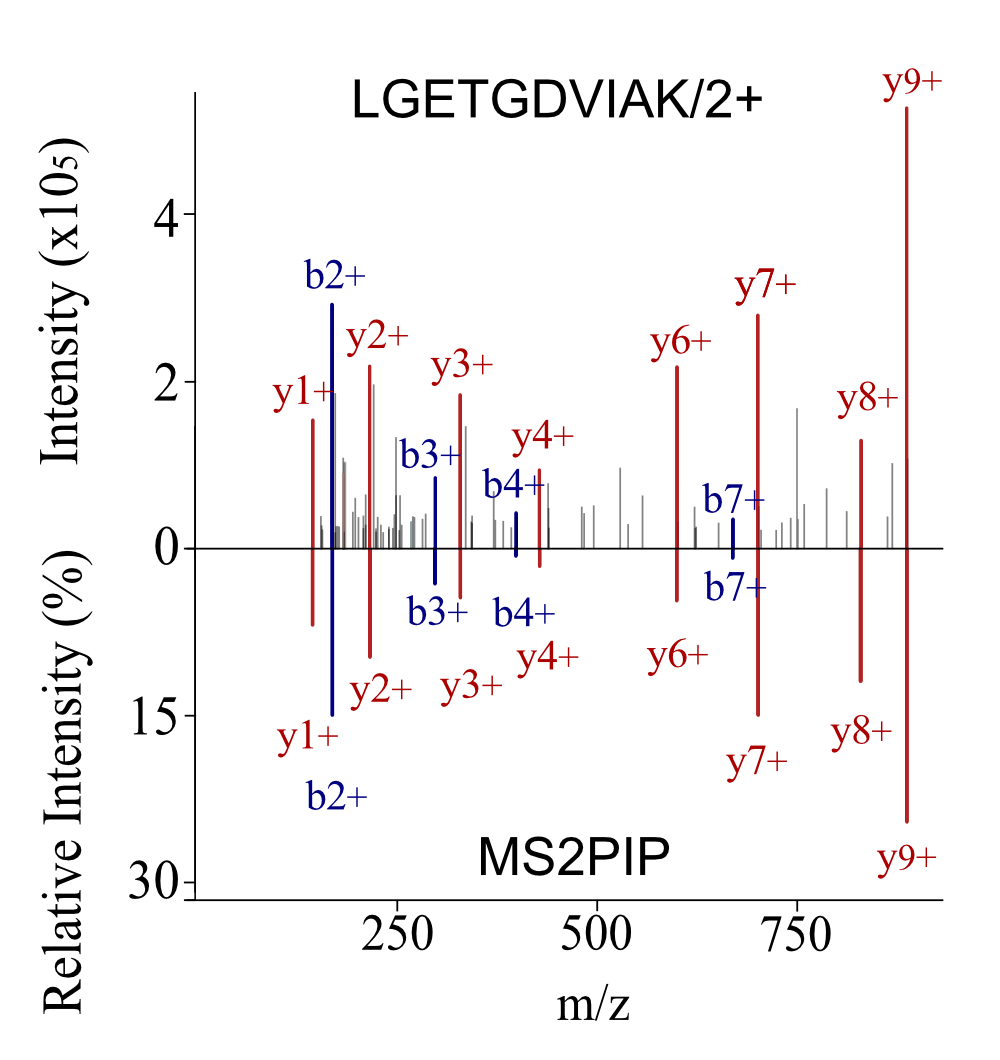

Supplement: uqac005_Supplemental_Files [file uqac005_supplemental_files.zip › SuppFig7.tiff]

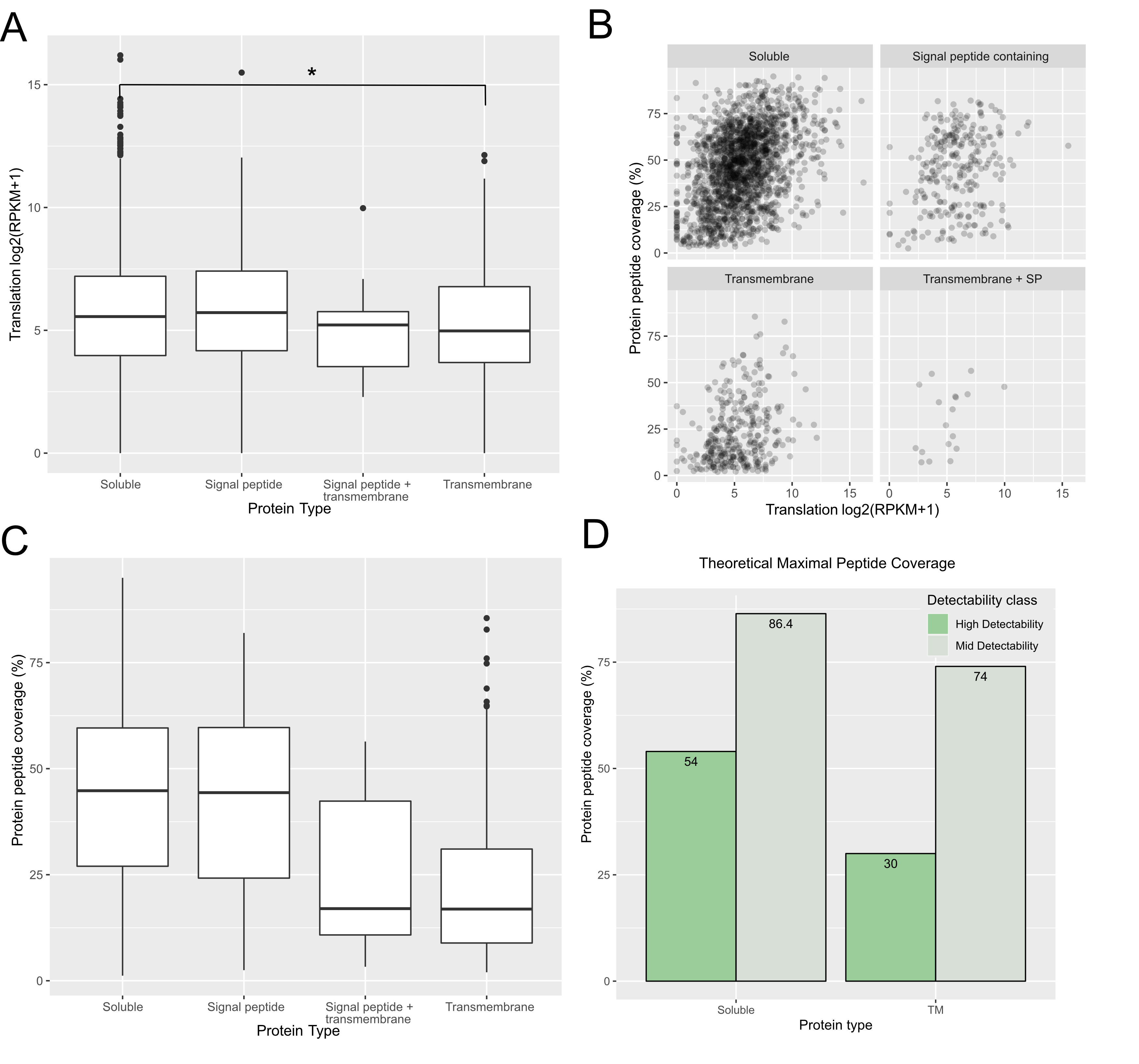

Supplement: uqac005_Supplemental_Files [file uqac005_supplemental_files.zip › SuppFig8.tiff]
